# Thoracic Hemisection in Rats Results in Initial Recovery Followed by a Late Decrement in Locomotor Movements, with Changes in Coordination Correlated with Serotonergic Innervation of the Ventral Horn

**DOI:** 10.1371/journal.pone.0143602

**Published:** 2015-11-25

**Authors:** Anna N. Leszczyńska, Henryk Majczyński, Grzegorz M. Wilczyński, Urszula Sławińska, Anna M. Cabaj

**Affiliations:** 1 Nencki Insitute of Experimental Biology, PAS, Warsaw, Poland; 2 Nałęcz Institute of Biocybernetics and Biomedical Engineering, PAS, Warsaw, Poland; National Institutes of Health / NICHD, UNITED STATES

## Abstract

Lateral thoracic hemisection of the rodent spinal cord is a popular model of spinal cord injury, in which the effects of various treatments, designed to encourage locomotor recovery, are tested. Nevertheless, there are still inconsistencies in the literature concerning the details of spontaneous locomotor recovery after such lesions, and there is a lack of data concerning the quality of locomotion over a long time span after the lesion. In this study, we aimed to address some of these issues. In our experiments, locomotor recovery was assessed using EMG and CatWalk recordings and analysis. Our results showed that after hemisection there was paralysis in both hindlimbs, followed by a substantial recovery of locomotor movements, but even at the peak of recovery, which occurred about 4 weeks after the lesion, some deficits of locomotion remained present. The parameters that were abnormal included abduction, interlimb coordination and speed of locomotion. Locomotor performance was stable for several weeks, but about 3–4 months after hemisection secondary locomotor impairment was observed with changes in parameters, such as speed of locomotion, interlimb coordination, base of hindlimb support, hindlimb abduction and relative foot print distance. Histological analysis of serotonergic innervation at the lumbar ventral horn below hemisection revealed a limited restoration of serotonergic fibers on the ipsilateral side of the spinal cord, while on the contralateral side of the spinal cord it returned to normal. In addition, the length of these fibers on both sides of the spinal cord correlated with inter- and intralimb coordination. In contrast to data reported in the literature, our results show there is not full locomotor recovery after spinal cord hemisection. Secondary deterioration of certain locomotor functions occurs with time in hemisected rats, and locomotor recovery appears partly associated with reinnervation of spinal circuitry by serotonergic fibers.

## Introduction

Lateral hemisection of the spinal cord at the low thoracic level is one of the models frequently used for testing new therapeutic strategies, which can be applied after spinal cord injury [1–3]. Although there are numerous inventive studies designed to improve locomotion *via* a variety of rehabilitation techniques or pharmacological agents after spinal cord hemisection, there is still a lack of precise data describing spontaneous locomotor recovery after such injury. There is common agreement that in the acute state after hemisection of the spinal cord at the low thoracic level, in rats with severe deterioration of the hindlimb, locomotor movement is present, followed by a substantial improvement in locomotor function. However, reports on the rate and the degree of this improvement remain inconsistent.

For example, according to Ballerman and Fouad [4], locomotion with consistent weight-supported hindlimb plantar stepping, which corresponds to a score of 14 on the BBB scale [5] is regained 14 days after hemisection. In experiments conducted by Redondo-Castro and coworkers [6], this degree of recovery was not achieved at all, and the maximal BBB score reached 50 days after hemisection is 12; this corresponds to frequent or consistent weight-supported plantar steps and no fore-hindlimb coordination. Arvanian, with others [7], conducted separate assessments for the hindlimbs ipsilateral and contralateral to the side of the lesion. The contralateral hindlimb function reached a score of 14 on the BBB scale 7 days after hemisection, while the ipsilateral hindlimb did not achieve that score until 35 days after hemisection. Contrary to these results, Edgerton’s group recently reported [8] that spontaneous recovery of locomotion in hemisected rats 3 weeks after the injury was characterized by “poor stepping” and “…partial trunk and tail support (was) necessary to enable effective weight bearing and coordination during stepping.” This degree of persistent impairment has not been reported in any other study using hemisection model. On the other end of the spectrum, results from hemisected rats show that locomotor performance returned to “normal” about 21 days after a lateral thoracic hemisection [9]. A detailed analysis of the locomotor effects of thoracic hemisection is warranted, and such an analysis is one of the aims of this study.

Some, but not all, of the discrepancies in the previous findings might be due to usage of the BBB scale. Although this method was created with the aim of achieving some standardization for evaluation of locomotor performance after spinal cord injury, and might be useful, especially in the first days after hemisection, it may not be sufficient to assess some subtle changes of the locomotor pattern, such as intra- and interlimb coordination and their relation to locomotor speed. Moreover, as Rossignol and colleagues have pointed out [10], locomotor function is not restored in a linear manner, as the BBB score assumes. Despite these differences in the rate and the degree of locomotor recovery, there is agreement that, 2–3 weeks following hemisection, locomotor performance reaches a plateau, and that after this period of time no robust improvement of locomotion is observed [4,7]. Unfortunately, none of the previous studies on locomotor recovery following hemisection of the spinal cord in rats involved experiments that were conducted longer than 6 to 10 weeks after injury, and not much is known about locomotor performance several months after injury. As pointed out by Dietz [11], there is a need for chronic animal models, which allow for the detection of neuronal changes after a long period of time to assess the damage to the supraspinal fibers and allow for the development of appropriate rehabilitation techniques. To provide insight into this problem, we evaluated the effects of thoracic hemisection over the course of a 5-month-period, which is a time after injury that may be considered as a chronic stage of injury.

It is also known that one of the key elements in the process of producing and restoring locomotion after spinal cord injuries is serotonin [12–15]. Embryonic grafts that contain serotonin-rich regions of raphe nuclei, when transplanted below the area of injury, can improve locomotor function restoration after complete spinal cord injury in rats [16–21]. It was also shown that restoration of serotonin innervation in the lumbar spinal cord occurs robustly during the 4 weeks after thoracic hemisection [22] and is correlated with recovery of locomotor abilities [9]. Thus, the aim of our study was to investigate changes in locomotor performance over time, into the chronic stage, after a low thoracic lateral hemisection of the spinal cord and to examine whether these changes are related to the re-growth of serotonergic axons in the ventral horn on the lumbar spinal cord level.

## Material and Methods

### Subjects

The experiments were performed on 45 female Wistar rats, 3-months old (250–300 g), at the beginning of the experiments. The animals were kept in separate cages in a room with 12:12 dark/light cycles. All of the procedures were conducted with the approval of the First Ethics Committee for Animal Experimentation in Poland according to the principles of experimental conditions and laboratory animal care of the European Union and the Polish Law on Animal Protection.

The experiments started from the time of spinal cord hemisection and lasted for 5 months. Locomotor investigation was carried out before spinal cord injury (SCI) and every day during the first 2 weeks after hemisection and then 3 times per week up to the moment when recovery of locomotor performance reach a plateau (about 1 month after the surgery). In the following 2 months, locomotion was tested once a week, and then every other week up to the end of experiments (5 months after the lesion). Three to five rats were taken at consecutive time-points (1, 2, 3 and 4 weeks and then 2, 3 and 5 months) after hemisection to investigate the lesion extent and the length of regenerated serotoninergic fibers below the level of hemisection.

### Spinal cord hemisection

Spinal cord lateral hemisection was conducted under deep anesthesia (2 l/min of O_2_ + 2% Isoflurane and s.c. Butomidor 0.05 mg/kg b.w.). Laminectomy was performed over the Th9 vertebral segment (approx. 1.5–2.0 mm) at the thoracic level. The left half of the spinal cord was dissected at the Th11 level using fine watchmakers forceps. After SCI, the overlying muscle layers were closed using sterile sutures (Mersilk 5–0). The skin was closed with stainless-steel surgical clips. After surgery, the animals received a non-steroidal anti-inflammatory and analgesic treatment (s.c. Tolfedine, 4 mg /kg b.w.), and during the following 7 days, the animals were given antibiotics (s.c. Baytril 5 mg/kg b.w. and Gentamycin 2 mg/ kg b.w.).

### Implantation of EMG electrodes

For EMG recordings, bipolar electrodes were implanted in the flexor and extensor muscles of each limb (in the forelimbs: the *Biceps Brachii* (Bic) and *Triceps Brachii* (Tri) medial head; in hindlimbs: the *Tibialis Anterior* (TA) and *Soleus* (Sol) muscles) under Equithesin (0.35 ml/100 g b.w. administered i.p.) anesthesia (see [18] for details). The electrodes were made of Teflon-coated stainless steel wire (0.24 mm in diameter; AS633, Cooner Wire, Chastworth, CA, USA). The tips of the electrodes, with 1–1.5 mm of insulation removed, were pulled through a cutaneous incision on the back of the animal, and each of the hook electrodes was inserted into the appropriate muscle and secured by a suture [18,23]. The distance between the electrode tips was 1–2 mm. The ground electrode was placed under the skin on the back of the animal some distance from the hindlimb muscles. The connector with the other ends of the wires fixed to it was covered with dental cement (Spofa Dental, Prague, Czech Republic) and silicone (3140 RTV, Dow Corning) and secured to the back of the animal. During locomotion, both of the extensors (Tri and Sol) and one flexor (TA) have only one burst of activity in each step cycle (during the stance phase and the swing phase, respectively). The Bic muscle presented biphasic activity, and thus, only bursts present during the swing phase were analyzed.

The locomotion was examined using two methods: EMG recordings and the CatWalk Gait Analysis System. For the EMG recordings, the procedure for the examination of locomotion of freely moving rats was based on a shelter-seeking paradigm. Rats were pre-trained to walk on a horizontal runway 2.5 m long and 12 cm wide with an illuminated start platform at one end and a darkened goal box at the other (for details see [23]). For CatWalk analysis, the rats were taught to move on a 1.5 m long by 20 cm wide runaway in the dark. One experimental session using both methods consisted of 10 passes. Runs during which the animals stopped or changed their speed rapidly were not subjected to further analysis. EMG analysis was performed to observe changes of temporal parameters of locomotion, while the CatWalk analysis was performed to analyze changes in spatial parameters.

During the first and second week after hemisection, locomotor movements were very unstable, and runway and CatWalk trials were not possible. Therefore, locomotion was examined using a treadmill.

### Analysis of temporal parameters of locomotor abilities using EMG recordings

EMG activity of selected muscles recorded during locomotion was filtered (0.1 to 1 kHz band pass), digitized and stored on a computer (2 kHz sampling frequency) using the Winnipeg Spinal Cord Research Centre Data Capture System. The locomotor EMG pattern was analyzed using custom software (http://www.scrc.umanitoba.ca/doc/).

#### Polar Plot analysis of inter- and intralimb coordination

Intralimb coordination (between the flexor and extensor of the same hindlimb, i.e. l Sol-TA and r Sol-TA), and interlimb coordination (between the flexors or between the extensors of both hindlimbs, i.e. l-r TA or l-r Sol) were determined using a polar plot analysis [24–27], as described previously [14,19,28]. Briefly, the rectified and integrated EMG records of the same periods of rhythmic activity were normalized to the step cycle with the onset of activity in the first muscle of each analyzed pair. The phase position of the polar-plot vector reflects the mean phase shift, i.e. the synchrony or the alteration of analyzed EMG bursts onsets. The length of the polar-plot vector reflects the ***r***-value (ranging from 0 to 1), which reveals the concentration of phase shifts around the mean phase shift. Rayleigh’s circular statistical analysis test was applied to determine if coupling of burst activity was present. If the ***r***-value was greater than the critical Rayleigh’s value for a given *p*-value, then coupling of burst activity was present and the locomotor movements were considered to be coordinated.

#### The fore-hindlimb coupling ratio (CR)

During spontaneous locomotion, at relatively constant speed intact rats exhibit a one-to-one relationship between the consecutive stepping of the fore- and hindlimbs. The number of steps produced by the forelimbs and the number of steps produced by the hindlimbs in a given time range is equal. In the same manner, in a given time range, the number of EMG bursts produced by hindlimb muscles is equal to the number of corresponding bursts produced by forelimb muscles. In contrast, after the lesion, when the animals lose body weight support and the ability to perform hindlimb plantar stepping and move using only their forelimbs, the analysis of muscle bursts allows for a comparison of the activity cycles in the fore- and hindlimbs. Here, we introduce a new parameter (the fore-hindlimb coupling ratio—CR), which characterizes the relationship between the fore- and hindlimb rhythms during locomotion. The CR was calculated as the ratio of the number of bursts of a forelimb muscle to the number of bursts of a selected hindlimb muscle within a run, containing 10–15 forelimb cycles. When the CR ratio was different from 1, this indicated uncoupled fore-hindlimb muscle activity. Four CRs were calculated for 4 pairs of muscles: l TA–Bic; r TA–Bic; l Sol–Tri and r Sol–Tri. Shortly after the spinal cord hemisection these fore-hindlimb coupling ratios were significantly impaired.

### Assessment of spatial gait parameters using the CatWalk Gait Analysis System

CatWalk Gait Analysis System allowed us to measure the following spatial parameters: 1) base of support (BOS) defined as the distance between the lines parallel to the direction of rat locomotion at points marked by the center of the paw prints for the fore- and hindlimbs; 2) hindlimb abduction (HA) defined as the distance between the lines that are parallel to the direction of the rat locomotion and across the center of the fore- and hind footprints and measured in a given pair of steps; 3) relative print distance (RPD) was defined as the distance between the lines that were perpendicular to the direction of rat locomotion and across the center of the fore- and hind footprints and measured in a given pair of steps. For all measurements, the center of the footprint was defined as the center of mass of the print. Moreover, with CatWalk the mean speed of a given run was calculated as a mean of stride length divided by step cycle duration. Speed was calculated individually for each limb and the rat velocity was calculated as a mean of four speeds.

### Serotonin fiber distribution around motoneurons

After completing all of the behavioral and EMG data rats were euthanized with an overdose of pentobarbital (i.p., 150 mg/kg) and perfused transcardially with the 40 ml of cold (4°) prefixative solution (0.9% NaCl (Polfa), 0.1% sodium nitrite (Sigma-Aldrich), 0.2% heparine (Polfa) and 0.5 M PBS (Takara Bio Inc)) to washout the blood, followed by 300 ml of cold PFA solution (4% paraformaldehyde (PFA), Sigma-Aldrich) and 0.2% picric acid (Sigma-Aldrich) in 0.5 M sodium phosphate buffer (PBS) for tissue fixation. The spinal cords were dissected out, postfixed in PFA solution for 1.5 h and then cryoprotected with 30% sucrose (Poch) solution with 0.04% sodium azide (Sigma-Aldrich) and kept at 4°C for at least one week. Then, the L4-L6 level of each spinal cord was isolated, embedded in freezing medium (Jung Tissue, Leica) and frozen on dry ice. The specimen was then placed in a cryostat at -20°C for 30 min. Fifty μm coronal sections were cut and mounted onto microscope slides (SuperFrost Ultra Plus, Thermo Scientific). The specimen was then dried and rinsed 3 times for 10 min with PBST (0.5 M PBS, 0.3% TritonX-100 (Thermo Scientific)). The slices were then incubated with primary antibody (rabbit anti-serotonin, Sigma-Aldrich), diluted 1:500 in PBST with 10% NDS (Normal Donkey Serum, Jackson Immunoresearch), overnight at 4°C. Then the primary antibody was washed with PBST and the slides were incubated with the secondary antibody (DyLight, 549-coinjugated, Donkey anti-rabbit, Jackson ImmunoResearch), diluted 1:100 in PBST with 10% NDS, for 4h at room temperature in the dark and in a humid chamber. After rinsing in PBST (3 × 10min), the sections were stained with Neurotrace Fluorescent Nissl Stains 488 (Molecular Probes), diluted 1:100 in PBST, for 40 min at room temperature. After rinsing in PBST (3 × 5min), the specimens were dried and covered with Vectashield Medium (Hard Set Mounting Medium, Vector).

### Anatomical verification of the lesion site

The level of the spinal cord containing the lesion (Th10/11) was carefully dissected, embedded with freezing medium and frozen on dry ice. The specimen was placed in a cryostat at -20°C for 30 min. Then, 40 μm coronal sections were cut, mounted on microscope glasses, dried and rinsed with 0.3% PBST (3 *×* 10min). Next, the samples were incubated with a primary antibody against neurofilament protein (Monoclonal Mouse Anti-Human Neurofilament Protein, Dako), diluted 1:200 in PBST with 5% NDS, at 4° overnight. The following day the slides were washed with PBST and then incubated with the secondary antibody (Donkey Anti-Mouse IgG, Invitrogen), diluted 1:200 in PBST with 5% NDS, for 2 h at room temperature in the dark. Nissl bodies were visualized by incubating the slides with Neurotrace Fluorescent Nissl Stains (Molecular Probes), diluted 1:100 in PBST, for 1h in the dark. The slides where then washed and the specimens dried and covered with Vectashield Medium. All of the results presented show data from hemisected animals that were verified to have no more than 1% of the spinal cord matter preserved in the ipsilateral half of the spinal cord, as well as no more than 1% of the contralateral half of the spinal cord affected by the surgical procedure.

The specimens were examined under a Nikon Optiphot-2 epifluorescence microscope equipped with a digital camera (QICAM, 12-bit, Qimaging). The photomicrographs were obtained using a 10× magnification with Image Pro Plus v. 4.0 software. The same camera and microscope settings were always used. Quantitative analysis was conducted using ImageJ software (http://rsb.info.nih.gov/ij/) with the relevant plugins necessary to perform skeletonization measurements (AnalyzeSkeleton, author: Ignacio Arganda-Carreras http://fiji.sc/AnalyzeSkeleton). In our study, skeletonization was used to measure the length of the serotonergic fibers. Using this method, the shape of a fiber was thinned to a width of a single pixel, which was positioned equidistant to the boundaries of the analyzed fiber. The total count of pixels represents the total length of the serotonergic fibers.

### Statistical analysis

Statistical analyses were carried out using one-way ANOVA, two-way ANOVA or MANOVA tests. First, we confirmed that the data was characterized by a normal distribution (with a Shapiro-Wilk test) and verified the homogeneity of the variance (with respect to MANOVA using Box M tests with respect to ANOVA—Cochran C, Hartley, Barlett test). Tukey HSD or Fisher LSD *post-hoc* tests were used. If the assumptions of normality or homogeneity of variance or both were violated, then the Kruskal-Wallis test was used. For correlation analysis, a Pearson’s correlation test was used.

## Results

### Spinal cord lesion

Inspection of the coronal sections of the spinal cord tissue under a light microscope revealed that a complete left hemisection was present in all of the animals included in this paper. Most of the preserved tissue was confined to the right side of the spinal cord. All of data presented in this paper were collected from hemisected animals verified to have no more than 1% of the spinal cord matter preserved on the ipsilateral half of the spinal cord, as well as no more than 1% of the contralateral half of the spinal cord affected by the surgical procedure.

### General observations

During the first two days after hemisection, the rats were paraplegic, and able to move using their forelimbs to drag their body and hindquarters. The left hindlimb (LH), ipsilateral to the lesion side, was passively extended and mostly paralyzed with its foot lying on its dorsal side. The right hindlimb (RH), contralateral to the lesion side, was also passively extended, but more often it was bent at the knee and lying under the rat’s belly with the dorsal side of its foot down. Small movements were observed at the knee or hip joints. In subsequent days, hindlimb locomotor performance gradually recovered. In the second to fourth day after the lesion, progressive recovery of RH locomotor functions was present. The right foot was often placed on its plantar surface and occasional body weight support was seen. In the LH, locomotor recovery was more limited and only occasional movements in the hip, knee and ankle joints occurred. Around the fifth day after hemisection, a regular RH plantar foot position was observed in most of the rats, but body weight support was only present sporadically. LH plantar stepping occasionally occurred in more than half of the hemisected animals, but body weight support was still missing. On the seventh day after hemisection, RH plantar stepping was present in all rats, although difficulties with full body weight support present. Eleven days after hemisection, plantar stepping was present in both hindlimbs, but due to intermittent LH body weight support, locomotion was very unstable, and the animals often fell to the left. When plantar stepping was present, both hindlimbs were abducted and the hindlimb base of support was much wider than in intact animals. These abnormalities persisted to the end of the study and were quantified in the CatWalk test (see Base of support (BOS) and Hindlimb abduction (HA) in the Results). Regular and sustained locomotion was regained in the majority of animals 14 days after lesion and thereafter the animals were tested using the CatWalk. It is important to underline that, in contrast to another study, [8] after day 14, all of the hemisected animals did not need assistance during locomotion for the remainder of the experimental period, and the animals moved with stable body weight support, plantar stepping and toe clearance.

### Changes in temporal locomotor parameters after spinal cord hemisection

**Fig 1:**
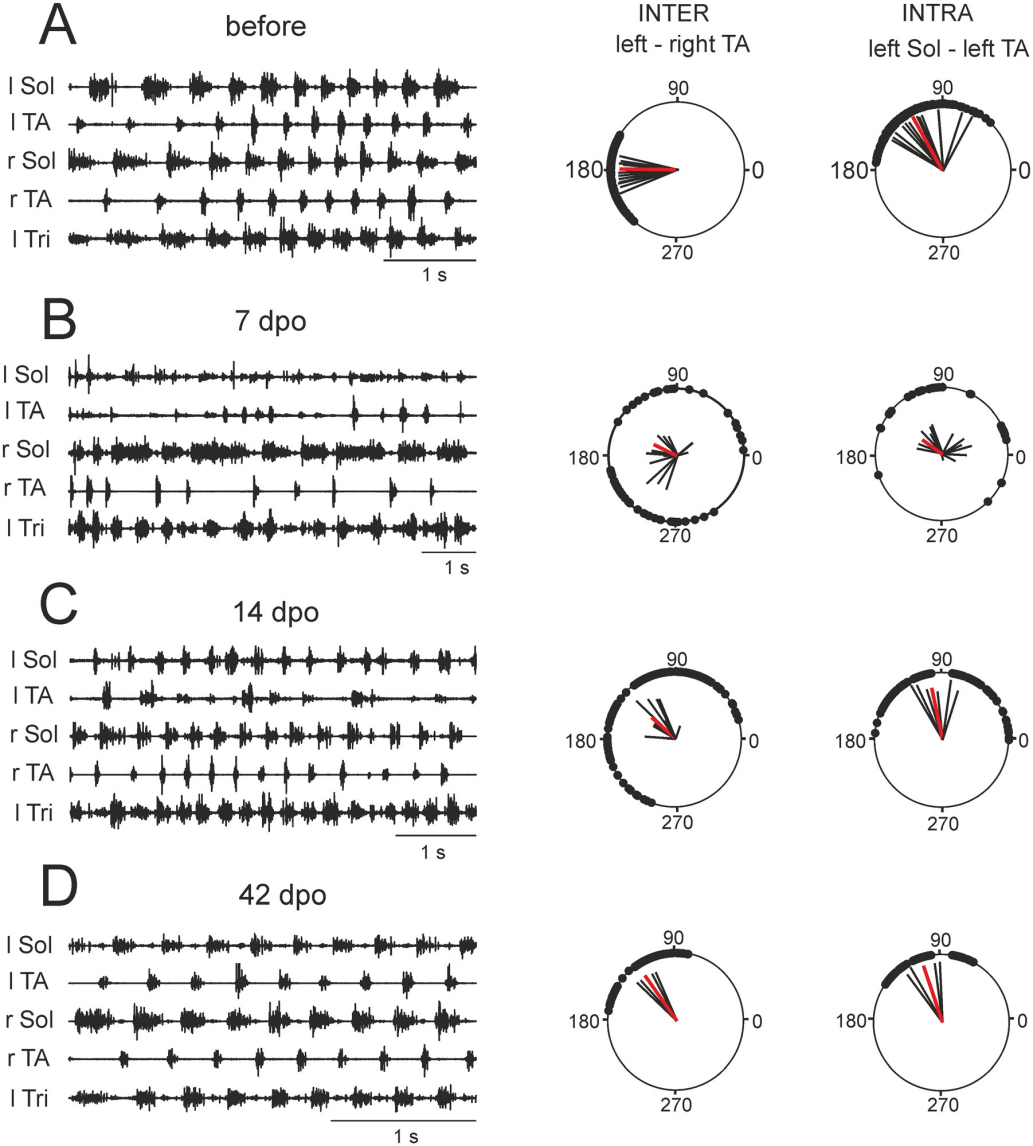
Comparison of EMG activity of the forelimb and hindlimb muscles. EMG activity during locomotion of a representative rat before (A), 7 days (B), 14 days (C) and 42 days (D) after spinal cord hemisection. Interlimb coordination (onset of EMG activity in the left TA (l TA) with respect to the contralateral right TA (r TA)) and intralimb coordination (onset of EMG activity in the left extensor (l Sol) with respect to the left TA (l TA)) for these time points are shown in the left and right polar plots, respectively. Zero corresponds to the onset of activity in the right TA muscle, and the positions of the filled black circles indicate the times of onset of activity in the left TA*—*interlimb coordination (left panel) or the onset of activity in the left Sol *-*intralimb coordination (right panel) in the relation to the onset of activity in the left TA muscle. The black lines each represent data from one animal, and the red line represents the global mean for the group. l Tri—left *Triceps*; l/r Sol—left and right *Soleus* and l/r TA—left and right *Tibialis Anterior*.

To investigate the effects of spinal cord hemisection on locomotor performance at different time points after injury EMG activity of the forelimb and hindlimb extensor and flexor muscles was recorded. These measures were used to establish changes in the temporal parameters describing the quality of limb movements. Our analysis revealed that a dramatic alteration in hindlimb locomotor abilities, induced by spinal cord hemisection, was always associated with a substantial alteration of hindlimb muscle activity. Fig 1 presents examples of EMG activity recorded during locomotion along the runway from the hindlimb muscles (left/right Sol and TA muscles) before and after spinal cord hemisection. During regular locomotor activity in intact rats, Sol and TA were active in a rhythmic alternating pattern, according to a regular step cycle. The Sol muscle, as an extensor and antigravity muscle, was active during the stance phase of each step cycle; while the TA muscle, as a dorsiflexor of the ankle joint, was active during the swing phase (Fig 1A). During the first few days after injury, EMG activity of Sol and TA muscles was limited when the animals were moving around the experimental box or the runway (Fig 1B). In subsequent days, when hindlimb locomotor performance gradually recovered after the spinal cord lesion, a progressive improvement in EMG recordings was present. Thus, starting from the 14^th^ day after the lesion, regular and sustained locomotion was associated with a regular rhythmic EMG activity (Fig 1C and 1D). The effects of hemisection on intra- and interlimb coordination are illustrated in the right panels of Fig 1.

#### Fore-hindlimb coordination (coupling ratio—CR)

Within the first week locomotor movement is confined largely to the forelimbs. Therefore, we investigated movement of the forelimbs and coordination of this activity with hindlimbs. Various methods for scoring locomotor performance, described in the literature, rely on the achievement of fore-hindlimb coordination. Some of these methods, like the BBB [5]method, use video recordings that provide limited temporal precision. Thus, in our investigation, where spinal cord hemisection produced an obvious lack of coupling between fore- and hindlimb movement, we used EMG burst activity, which is present even when hindlimbs are not successfully stepping, to analyze limb coordination in a more precise way. Based on EMG burst activity, we calculated a parameter that we termed the “coupling ratio” (CR). This is the ratio of the number of bursts of a selected hindlimb muscle to the number of bursts of a forelimb muscle during a defined period of locomotion.

**Fig 2:**
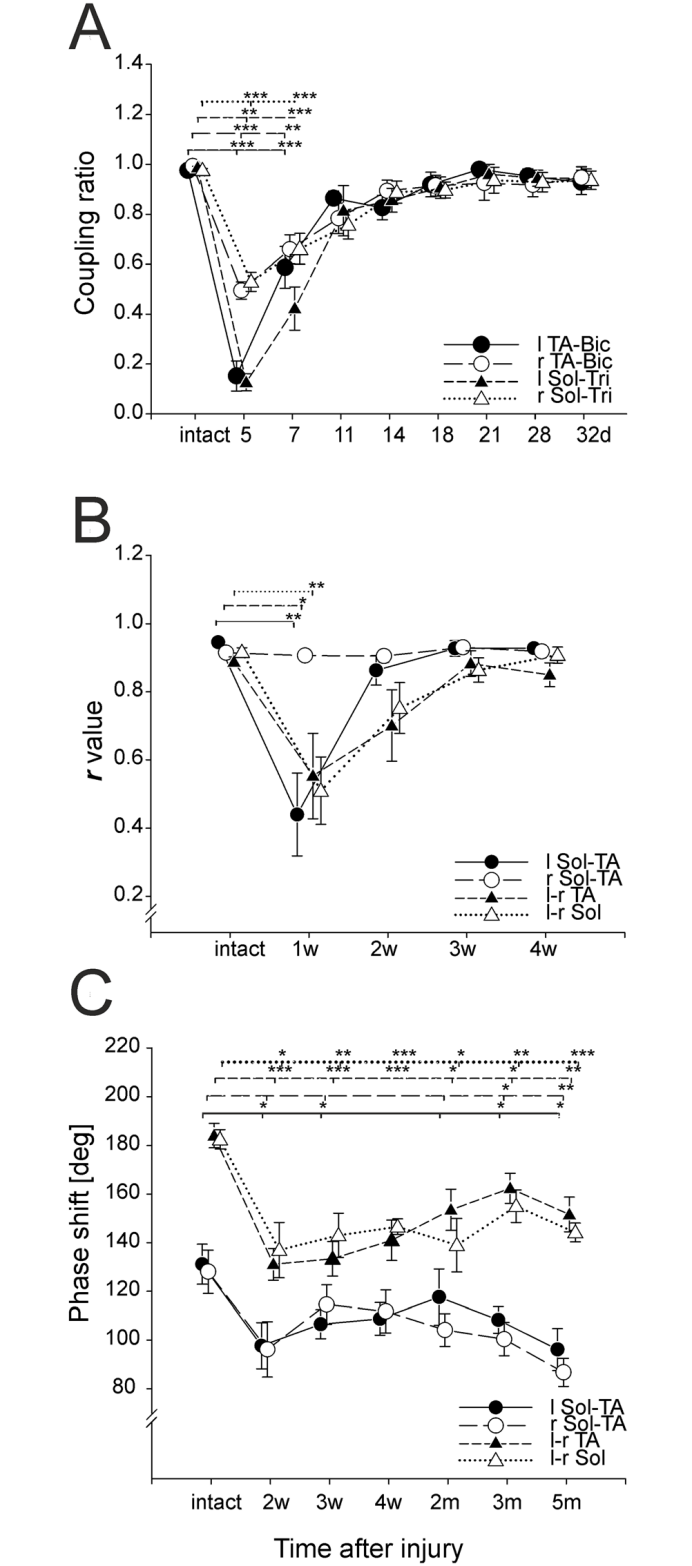
Changes in temporal parameters induced by spinal cord hemisection. (A) Time course showing the changes in fore-hindlimb coordination (coupling ratio represented as the mean±SEM) of fore- and hindlimb flexor (l and r TA-Bic) and extensor (l and r Sol-Tri) muscles post hemisection. (B) Time course showing the changes in interlimb (l TA—r TA; l Sol—r Sol) and intralimb (l Sol—l TA; r Sol—r TA) coordination post hemisection (***r***-values are represented as the mean±SEM). (C) Time course showing the changes in the phase shift in interlimb (l TA—r TA; l Sol—r Sol) and the intralimb (l Sol—l TA; r Sol—r TA) coordination post hemisection (***r***-values are represented as the mean±SEM). Abbreviations: d—days post operation, w—weeks post operation, m—months post operation, * − *p*<0.05, ** − *p*<0.01, *** − *p*<0.001.

During unrestrained locomotion in intact animals there is a strict one-to-one step correlation in fore-hindlimb stepping which is expressed as an alternating pattern of EMG bursts (Fig 1A). Thus, the number of EMG bursts produced by the hindlimb muscles is similar to the number of corresponding bursts produced by forelimb muscles. As expected, during locomotion in intact rats CR was close to 1 (0.959 ± 0.013 and 0.981±0.09 for l TA-Bic and r TA-Bic, respectively) (Fig 2A; S1 Table).

In the first two days after hemisection, the animals moved primarily using their forelimbs and showed minimal hindlimb EMG bursts during locomotion on the treadmill, either in the muscles of the hindlimb ipsilateral to the lesion side or in the muscles of the contralateral hindlimb. However, in the subsequent days, bursts of EMG activity of hindlimb muscles during locomotion gradually started to appear.

Our analysis showed a significant decrease in the CR values for all four of the muscle pairs as a result of spinal cord hemisection. Kruskal-Wallis analysis revealed that hemisection produced a significant decrease in the CR values for all four of the muscle pairs (l TA-Bic: H = 44.5, *p*<0.001; r TA-Bic: H = 39.6, *p*<0.001; l Sol-Tri: H = 33.5, *p*<0.001; r Sol-Tri: H = 33.5, *p*<0.001). Multiple comparisons analysis showed differences at 5 and 7 days post-operation with respect to the CR values of all four of the muscle pairs (see Fig 2A). On day 5 post hemisection, the number of hindlimb muscle EMG bursts was much smaller than before hemisection, especially for the LH muscle (CR values were 0.152±0.060 and 0.126±0.034 for l TA-Bic and l Sol-Tri, respectively). For the RH muscle, CR values were greater than for the LH muscle (0.495±0.034 and 0.529±0.038 for r TA-Bic and l Sol-Tri, respectively), but still much smaller compared to intact animals. These CR values show that the number of EMG bursts in hindlimb muscles was smaller compared to forelimbs. Seven days after the lesion, CR values increased but were still smaller than before hemisection (0.587±0.084 (l TA-Bic), 0.423±0.087 (l Sol-Tri), 0.660±0.058 (r TA-Bic) and 0.662±0.062 (r Sol-Tri)). There was no difference between the CR values for all four pairs of muscles. By day 11, the mean CR values increased and were not markedly different from what was calculated before hemisection (0.865±0.049, l TA-Bic; 0.814±0.101, l Sol-Tri; 0.784±0.061, r TA-Bic and 0.757±0.056, r Sol-Tri). Thirty-two days after hemisection, the CR values approached 1 (0.928±0.049, l TA-Bic; 0.944±0.038, l Sol-Tri; 0.947±0.049, r TA-Bic and 0.936±0.037, r Sol-Tri). The CR values remained at a similar level until the end of the study (5 months post hemisection).

#### Flexor-extensor (f-e) and left-right (l-r) hindlimb coupling–*r*-value

In our study, intralimb (f-e) and interlimb (l-r) hindlimb coupling was investigated using a polar-plot analysis (Fig 1, right panels). In intact rats, during locomotion, EMG bursts of muscle activity occurred regularly, and the ***r***-values indicated a high degree of coordinated activity in the muscle pairs (0.888±0.015 for l-r TA and 0.946±0.013 for l Sol-TA with *p*<0.001), (Figs 1A right panels and 2B). Shortly after the lesion, when animals moved with their forelimbs and no or rare movements in a few hindlimb joints were observed, hindlimb muscle activity occurred sporadically and the onset of EMG bursts was uncoordinated, as demonstrated by small ***r***-values (see Figs 2B and 1B, right panels). Then, within several days hindlimb muscle activity returned to normal, but the phase shift between the burst muscle activities remained altered (see below).

Kruskal-Wallis analysis revealed that hemisection produced a significant effect on the ***r***-values of l–r Sol interlimb coordination (H = 16.6, *p* = 0.002), l-r TA interlimb coordination (H = 15.3, p = 0.006 and l Sol-TA intralimb coordination (H = 12.4, *p* = 0.01) but not on r Sol-TA intralimb coordination (H = 6.4, *p* = 0.52). This indicated that the ankle flexor-extensor regularity in the limb contralateral to the lesion side was not affected by hemisection. Multiple comparison tests showed that a significant alteration of inter- and intralimb coordination occurred one week after hemisection (l-r Sol: *p*<0.01, *r* = 0.510±0.099; l-r Tib: *p*<0.05, *r* = 0.546±0.125, l Sol-TA: *p*<0.01, *r* = 0.440±0.121). Smaller ***r***-values reflected the irregularity in coordination of burst activity in the analyzed muscles. No further statistically significant differences were observed at later time points, indicating that after the 2^nd^ week post-hemisection the regularity of EMG bursts was restored (***r***-values 0.701±0.105 (l-r TA) and 0.905±0.018 (r Sol-TA)). Four weeks after hemisection the ***r***-values were 0.850±0.035 (l-r TA) and 0.928±0.01 (l Sol-TA) and remained at these values to the end of the study (5 months) (Fig 2B; S2 Table).

#### Flexor-extensor and left-right hindlimb coupling—phase shift

Phase shift (the angle of the vector in the polar plot analysis showing the difference between the onset of EMG bursts in two muscles) was analyzed after spinal cord hemisection when the ***r***-value reached significance for all muscle pairs i.e. at the two week time point. Intralimb coupling, characterized by the phase shift between the f-e muscle onsets, did not change in either hindlimb after hemisection, while interlimb coupling, defined by the phase shift between the l-r extensor or the l-r flexor bursts, was different from intact animals up to 5 months after the lesion (compare the angles in the polar plots for inter- and intralimb coordination in Figs 1A–1D right panels and 2C; S3 Table).

Before spinal cord hemisection, phase shift of intralimb coordination was 131.1±8.2 (l Sol-TA) and 128.0±8.8 (r Sol-TA). One-way MANOVA did not reveal an effect of hemisection on phase shift of intralimb coordination for l Sol-TA or r Sol-TA: Wilks’ λ = 0.56, F(12, 64) = 1.75, *p* = 0.07. Two weeks after hemisection, phase shifts were 97.7±9.5 (l Sol-TA) and 96.2±11.4 (r Sol-TA), while five months after hemisection phase shifts were 96.1±8.6 and 86.8±5.7, respectively (Fig 2C).

In intact rats, the mean phase shift between l-r Sol and l-r TA (interlimb coordination) was 182.5±4.0° and 184±5.0°, respectively and as expected was influenced by hemisection (Fig 2C). One-way univariate ANOVA revealed that the l-r TA and l-r Sol mean phase shifts were changed after hemisection (F = 7.48, *p*<0.001, and F = 5.12, *p* = 0.001, respectively). A series of *post-hoc* Fisher’s LSD tests showed that, until the end of the experiment, the mean l-r TA phase shift was smaller compared to the value obtained before hemisection and was 131.1±6.6° (*p*< 0.001) 2 weeks after hemisection. In the following weeks phase shift gradually increased and after 2–3 months was significantly greater compared to the 2 weeks post lesion time point. However, this increase was followed by a later decrease in phase shift. Despite the temporary increase, l-r TA phase shift was smaller (*p*<0.01) after the lesion in comparison to the intact animals (*p*<0.01). Phase shift between the l-r Sol EMG burst onsets also was significantly smaller after hemisection and did not change significantly in the weeks after the lesion. Phase shift was smaller than before the lesion and at the second week post hemisection was 137.0±11.4° (*p*<0.001) and by 5 months was 144.2±3.9° (*p* <0.01). These results showed that phase shifts between burst onsets of interlimb coordination were not restored after hemisection.

### Recovery and subsequent decrement of spatial locomotor parameters after hemisection—CatWalk data analysis

**Fig 3:**
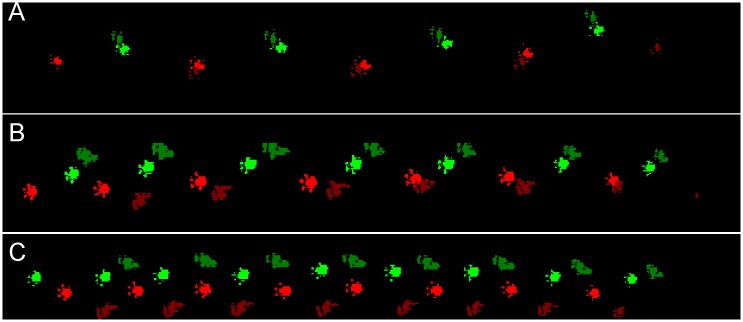
CatWalk foot prints examples before and after spinal cord hemisection. The left and right limb foot prints are shown in red and green, respectively. Darker shades represent hindlimbs and the lighter shades represent forelimbs. (A) intact rat, (B) 3 months after hemisection and (C) 5 months after hemisection.

**Fig 4:**
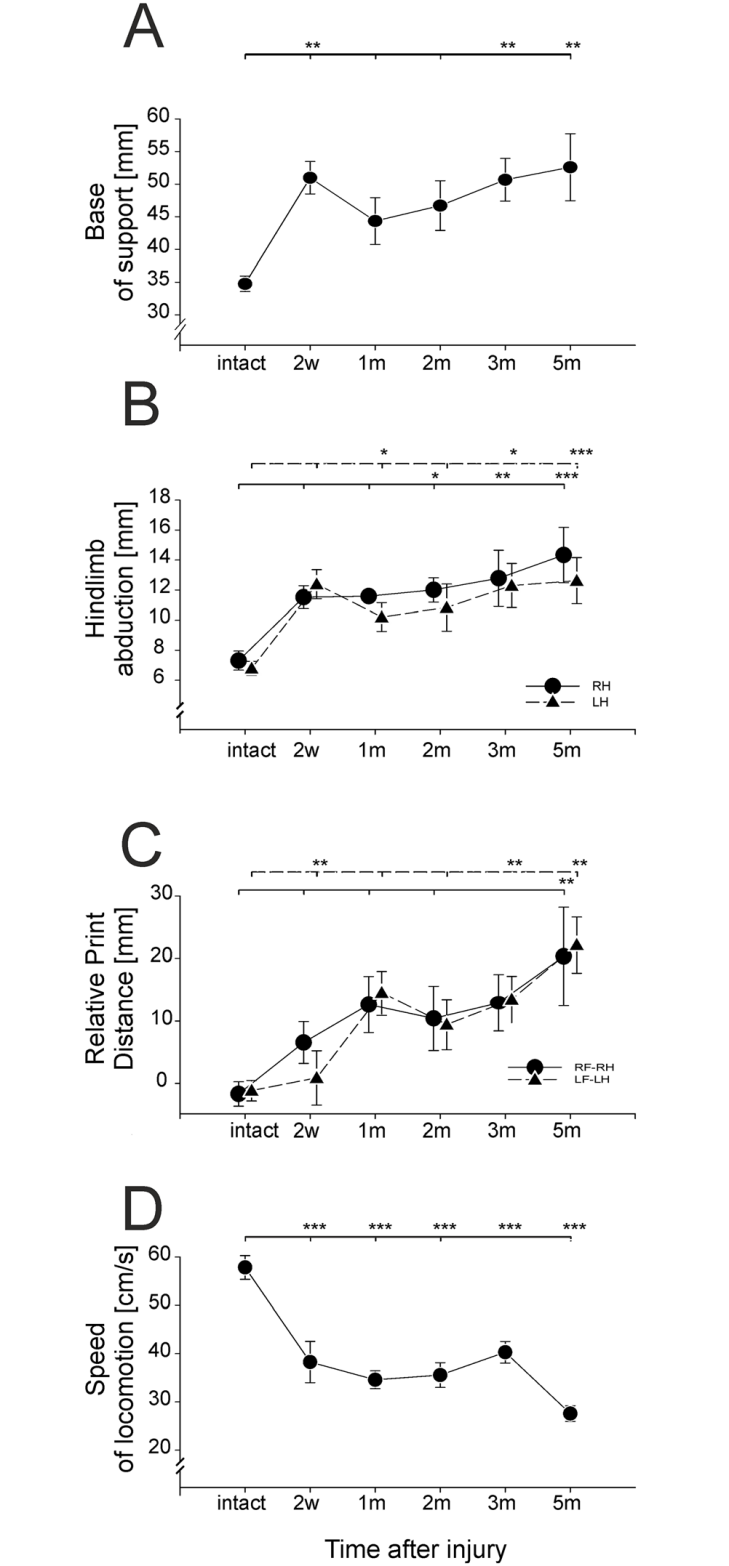
Changes in speed and spatial parameters induced by spinal cord hemisection. (A) Changes in the hindlimb base of support (BOS) before and at consecutive time points after hemisection (mean±SEM), (B) changes in hindlimb abduction (HA) before and at consecutive time points after hemisection (mean±SEM), (C) changes in the relative print distance (RPD) before and in the consecutive time points after spinal cord hemisection (mean±SEM), (D) changes in locomotor speed (V) before and at consecutive time points after hemisection (mean±SEM). Abbreviations: w—weeks post operation, m—months post operation, * − *p*<0.05, ** − *p*<0.01, *** − *p*<0.001.

CatWalk analysis was possible two weeks after hemisection, when the rats were able to engage in plantar stepping with both hindlimbs. An example from a single animal is shown in Fig 3. Before hemisection, the print distance between footfalls for the forelimbs and hindlimbs is very small, the base of support (BOS) is relatively small and no hindlimb abduction (HA) is present (Fig 4A and 4B). After injury there is a permanent change in print distance, BOS and HA (Fig 4A, 4B and 4C), and locomotor speed is reduced (Fig 4D). After 3 months, a secondary deterioration occurs in speed and print distance, when the rats begin to walk more slowly, with an increased BOS, HA and RPD. This could be due to a voluntary adaptation with the rat adjusting to the deficits associated with hemisection by adopting a wide-based gait.

#### Base of support (BOS)

In intact rats, the mean distance between the hindlimb footprints (BOS) was 34.8±1.2 mm (Fig 4A). A one-way ANOVA revealed that hemisection had a significant effect on the hindlimb BOS (F = 5.2, p = 0.001). A more detailed analysis, with Tukey HSD post-hoc comparisons, showed that two weeks after hemisection the BOS mean value dramatically increased (BOS = 51.0±2.5 mm) and was wider than before the lesion (p<0.001). In the following months, the BOS decreased and was not different from the value obtained before hemisection in the first and 2^nd^ month after surgery. However, from the 2^nd^ month after the lesion, the hindlimb BOS increased gradually and was greater than before surgery 3 and 5 months after hemisection, reaching 50.7±3.3 mm (*p*<0.001) and 52.6±5.1 mm (*p*<0.001), respectively. These results indicate that 3 months after hemisection a secondary deterioration of the hindlimb BOS occurred (Fig 4A; S4 Table). Increased BOS 3 and 5 months after the lesion was not an effect of a decrease in speed but rather an effect of late changes in the spinal cord neuronal network or a voluntary adaptation since, in intact rats BOS was independent of the speed of locomotion.

#### Hindlimb abduction (HA)

In intact animals, the mean abduction of the right and left hindlimb was not different (7.3±0.6 mm, and 6.8±0.5 mm, respectively) (Fig 4B; S5 Table). We measured abduction 2 weeks after hemisection, when animals could be tested in the CatWalk. The analysis revealed that, 2 weeks after the lesion abduction in both legs was almost 2 times greater than in intact animals. One month after the lesion left hindlimb abduction (l HA) dropped slightly, but the right (r HA) remained at the same level. In the following month, HA gradually increased, reaching values of 14.33±1.84 (r HA) and 12.62±1.53 (l HA). One-way univariate ANOVA revealed that hemisection affected r HA (F = 5.9, *p*<0.001) and l HA (F = 39.4, *p*<0.001). Tukey HSD *post-hoc* analysis showed that l HA was significantly greater than before the lesion two weeks (*p*<0.01), 3 months (*p*<0.01) and 5 months (*p*<0.01) after hemisection, and r HA was also significantly greater than before the lesion 2 months (*p*<0.05), 3 months (*p*<0.01) and 5 months (*p*<0.01) after hemisection. The persistence of a significant HA is consistent with the maintained increase in BOS up to 5 months after hemisection.

#### The relative print distance (RPD)

The relative print distance shows the reciprocal location of ipsilateral foot traces in a given pair of limb steps. In intact animals, the hind paws were positioned very close to the trace of the fore paws, and the mean right and left relative print distances were not different (-1.7±1.9 mm and -1.2±1.6 mm, respectively, Fig 4C). The negative value shows that the hind paw is positioned in front of the ipsilateral front paw trace. After the lesion, both of the RDPs were positive, indicating that the hind paws were placed behind the corresponding fore paw traces and gradually increased, reaching a plateau 1 month after the lesion. This trend did not change up to 3 months but then increased again, reaching values of 20.36±7.86 (right RDP) and 22.20±4.45 (left RDP) (Fig 4C; S6 Table).

One-way univariate ANOVA tests revealed that hemisection had an effect on the right RDP (F = 3.8, *p* = 0.009) and the left RDP (F = 7.3, *p*<0.001). The right RPD, contralateral to the lesion side, becomes significantly greater than in intact animals by the 5^th^ month after hemisection. (*p*<0.01), The ipsilateral left RPD was significantly greater than before hemisection starting from the 1^st^ month post lesion (*p*<0.05) and continuing through the 3^rd^ (*p*<0.05) and 5^th^ (*p*<0.001) months as revealed by the Tukey HSD *post-hoc* test. Thus, the absence an RPD recovery confirms the absence of recovery of normal locomotion after hemisection.

#### Locomotor speed (V)

Before hemisection, the rats moved along the CatWalk runway with a mean locomotor speed of 57.8±2.5 cm/s. One-way ANOVA revealed that hemisection had a significant effect on the speed of locomotion (F = 14.4, *p*<0.001). The detailed Tukey HSD *post-hoc* analysis revealed that, after hemisection, the mean speed was lower than before surgery through the whole experimental period (5 months) and did not change between the 2^nd^ week to the 3^rd^ month after the surgery (speed range was from 34.6±1.8 cm/s to 40.3±2.2 cm/s) but then dropped to 27.6±1.6 five months after hemisection (Fig 4D; S7 Table). At all of the analyzed time points, the mean speed of the intact animals did not change and was similar to the mean speed of the animals before surgery (data not shown). This indicates that the decrease in the speed after hemisection was a result of the SCI and not due to aging of the rats, the environmental condition in the home cages or experimental procedures. Therefore, locomotor speed is another parameter that, not only never recovered to pre-lesion values, but also deteriorated with time after the lesion.

#### Changes in serotonin innervation after spinal cord hemisection

**Fig 5:**
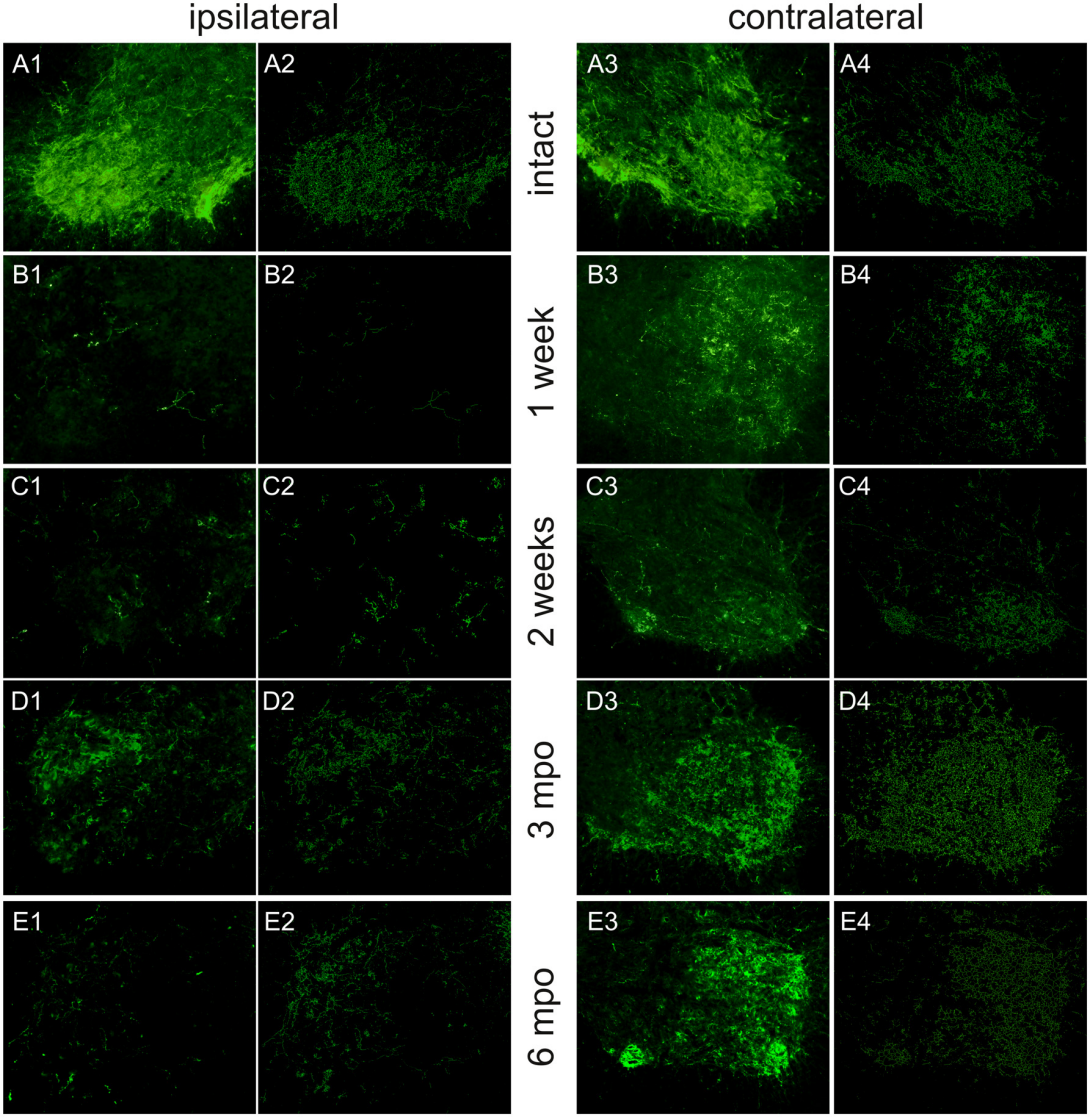
Immunolabeling of transverse spinal cord sections showing 5-HT-positive staining in intact rats and at different time points after hemisection. The representative images show the ventral horns below the lesion (L3-L5 level) in the ipsilateral (left) and contralateral (right) side to hemisection. (A) intact rat, (B) 1 week, (C) 2 weeks, (D) 3 months, (E) 6 months after the lesion. 1 and 3 –real images, 2 and 4 –images after skeletonization.

**Fig 6:**
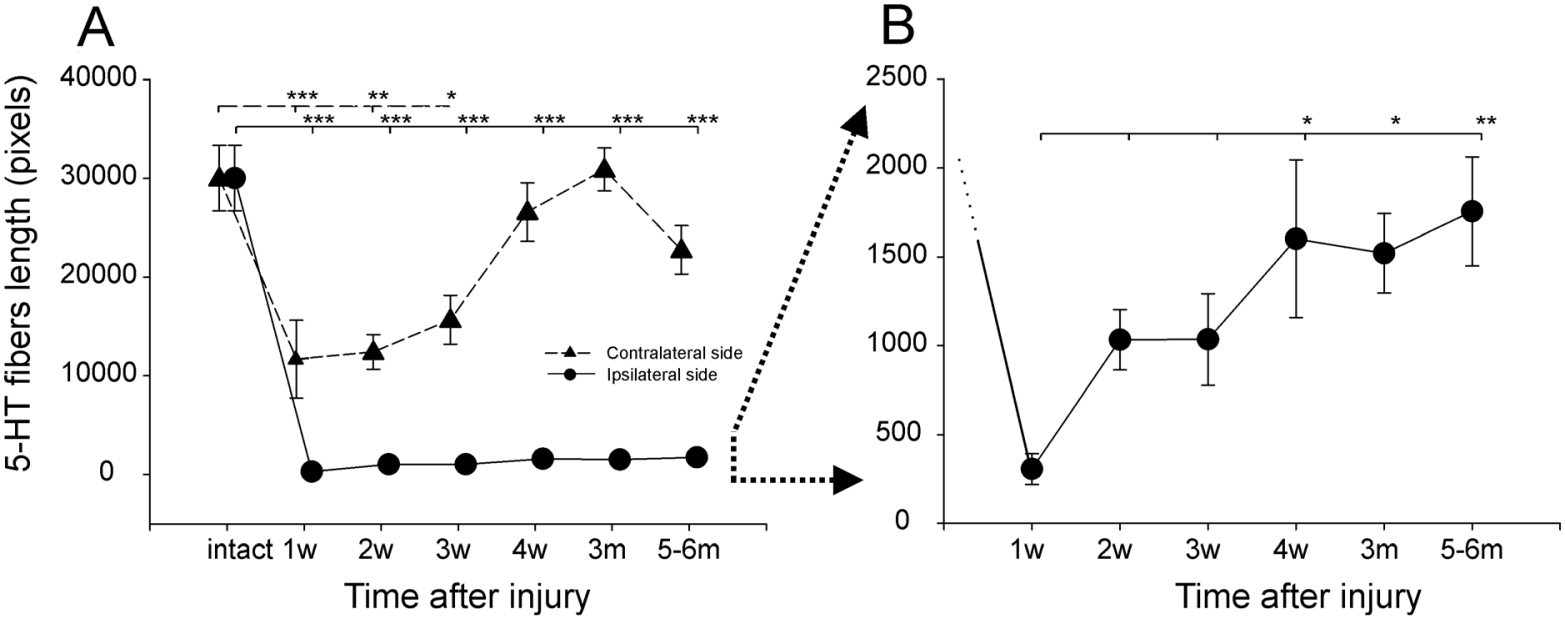
Serotonin fiber length. (A) Serotonin fiber length (in pixels) at the lumbar spinal cord level (below the area of hemisection) in the ipsi- and contralateral sides before and at consecutive time points after injury (mean±SEM). (B) Expanded scale of the diagram in (A) to show changes in serotonin fiber length (in pixels) in the lumbar spinal cord level (below the area of hemisection) in the ipsilateral side at consecutive time points after the injury (mean±SEM). Abbreviations: w—weeks post operation, m—months post operation. * − *p*<0.05, ** − *p*<0.01, *** − *p*<0.001.

We analyzed serotonin innervation in the ventral horn below the lesion, where the motoneurons supplying muscles of the hindlimbs are located, assuming that the concentration of the serotonin fibers in this region may be correlated with locomotor performance. In intact animals, the serotonergic fibers were evenly distributed in left and right ventral horns (Figs 5A1–5A4 and 6A). After hemisection, the number of 5-HT fibers reduced dramatically (Fig 6A). One week after lesion, serotonergic fibers on the left, ipsilateral side of the spinal cord were almost absent, while the number on the right side was reduced (Fig 5B4 and 5B5). In the following months, the number of the fibers increased and was almost equal to their numbers in intact animals on the right side contralateral to the lesion, while on the left side it remained reduced even 5–6 months after hemisection when the last verification of the 5-HT fibers was performed (Figs 5D1–5D4, 5E1–5E4 and 6; S8 Table).

To evaluate changes in serotonergic innervation in the spinal cord at different time points after hemisection, tissue specimens were assessed using a measure of serotonin fiber length on the right and left side of the lumbar spinal cord (L4-L5) in the ventral horns. To estimate the length of the 5-HT fibers, a skeletonization algorithm was used (http://fiji.sc/AnalyzeSkeleton). Our analysis shows that, in intact animals, serotonin fibers in the ventral horn of the lumbar spinal level are abundant and prominent (Fig 6A). The measurement performed using the skeletonization algorithm showed that the length of the serotonin fibers was about 30,000 pixels in each selected part of the ventral horn. After hemisection, fiber length decreased on both sides of the spinal cord. A one-way ANOVA showed that hemisection significantly affected the length of serotonergic fibers in this area both on the contralateral side of the spinal cord (F = 8.42, *p*<0.001) and the ipsilateral side (F = 72.41, *p*<0.001) (Fig 6A).

One week after hemisection, there was almost complete loss of immunoreactive fibers on the ipsilateral side of the lumbar spinal cord (Fig 6A). The mean length was 306±87 pixels and was about 100 times less than in intact animals (*p*<0.001). In the consecutive weeks, the length of the serotonergic fibers in the sample area gradually increased, and starting from the 4^th^ week after hemisection, the mean length was higher than one week after hemisection (*p*<0.05). Six months after injury, the mean number of serotonergic fibers was equal to 1756±307 pixels. Although this value was very low compared to the length of serotonergic fibers in intact animals, it was 6 times greater than one week after hemisection (*p*<0.01) (Fig 6B).

The mean length of the serotonergic fibers on the side of the spinal cord contralateral to the lesion also decreased, and one week after hemisection it was 11700±3946 pixels, which was about 60% smaller than in intact animals (*p*<0.005, Tukey *post-hoc* test). Starting in the 2^nd^ week after hemisection, the number of serotonergic fibers gradually increased but was still lower than in intact animals 2 weeks (*p*<0.01) and 3 weeks (*p*<0.05) after surgery. Starting from 1 month after the lesion, the mean number of serotonergic fibers on the contralateral side of the spinal cord was 26608±2952 pixels and was similar to the intact animals up to 6 months after hemisection (Fig 6A).

#### Correlation between serotonergic fiber length and the restoration of locomotion

To find if a relationship between the locomotor recovery and the restoration of serotonergic fibers exists, a Pearson’s correlation analysis was carried out. Our analysis did not show a relationship between serotonergic fiber length and the restoration of most locomotor parameters (S9 Table). However, we did find a significant correlation between the mean values of serotonergic fiber length observed in the consecutive time points after hemisection and the ***r***-values for inter- and intralimb coordination. The mean ***r***-value of the interlimb coordination between the Sol muscles of both hindlimbs (l-r Sol) correlated with: 1) the mean length of the serotonergic fibers on the ipsilateral side of the lumbar spinal cord (r = 0.95, p<0.005); and 2) the mean length of the serotonergic fibers on the contralateral side of the lumbar spinal cord (r = 0.87, *p* = 0.03). The ***r***-value of the intralimb coordination of the muscle activity of the ipsilateral limb (l TA-Sol) correlated with the fiber length on the ipsilateral side of the spinal cord (r = 0.98, *p* = 0.001). These findings are consistent with the observation that the action of serotonin on the spinal locomotor system is to promote intra- and interlimb coordination [29–33].

## Discussion

In this study, we describe the time course of locomotor recovery following a low thoracic lateral hemisection in rats from the first day up to 5 months post lesion using a battery of locomtor tests. We showed that immediately after hemisection there was a loss of hindlimb movements in the hindquarters, and at this stage the rats moved around the home cage using their forelimbs only. In the following days, the animals recovered gradually and regained stable four-limb locomotor abilities by the end of the first month after the injury. By that time, some of the locomotor parameters returned to values similar to that observed before the lesion, but most of them reached a plateau, with values showing decreased locomotor performance in comparison to the intact animals. Moreover, about 3 months after the lesion, further deterioration of locomotor ability could be seen, which was reflected by changes in several locomotor parameters. This phenomenon has not been previously described in the literature but is consistent with the findings in human locomotion in the chronic stages after spinal cord injury where exhaustion of locomotor activity is observed (reviewed by [34]). Previous studies on spinal cord hemisection in rodents have largely been limited to short-term recovery, but here, we extend the analysis to 5 months, which is comparable to chronic injury in humans. Our results also do not confirm previous reports that locomotion returns to “normal” after hemisection in rats [9]. Deficits persist in interlimb coordination, speed of locomotion, base of support, fore-hind foot print distance and left and right hindlimb abduction. While, the permanent locomotor deficits after hemisection are consistent with previous observations [1,6,7,35–41].

An important stage in the process of locomotor recovery is the moment when rats regain the ability to place their hind feet on the plantar surface with body weight support. Our investigations revealed that rats usually started to place their contralateral hind foot on its plantar surface on the 5^th^ day after hemisection and the ipsilateral hind foot on the 11^th^ day. This plantar stepping was associated with body weight support. However, locomotion was unstable, and the hindquarters partly collapsed quite often. By about day 14, quadrupedal locomotion became consistent enough to perform at least ten consecutive plantar steps in a run (essential for evaluating locomotor performance using the CatWalk system). These results are consistent with most other studies in which changes in hindlimb locomotor performance after hemisection were analyzed over time. In these studies, it was revealed that the spontaneous recovery of locomotor performance was different in the hindlimb ipsilateral and contralateral to the spinal cord hemisection side and that, about 7 days after the injury, plantar stepping with body weight support occurred in the contralateral hindlimb, while at that time no plantar stepping was observed in the ipsilateral side. At day 14, the locomotor performance of hemisected rats reached a plateau, which lasted to the end of the study (25–70 days) [1,4,7,9,41]. In a small number of studies recovery of consistent plantar stepping with body weight support was delayed beyond what we describe [39] or did not return at all [8]. In Shah’s studies rats needed partial trunk and tail support to perform weight bearing and coordinated locomotion. This poor locomotor performance in rats with spontaneous recovery of locomotion might be associated with larger lesion sizes than were used in our study. Moreover, Shah and colleagues [8] showed that training improved locomotor performance, and rats could locomote unassisted. Contrary to Shah’s results, Cloud and collaborators [35] demonstrated that training lasting from the 7^th^ to the 63^rd^ day after hemisection did not improve locomotor performance.

The initial loss of plantar stepping is a common phenomenon observed after various lesions of the spinal cord. Schucht and collaborators [42] showed that plantar stepping was present in rats after a near-total spinal lesion if only the white matter in the ventral columns was preserved. In contrast, preserving the whole dorsal columns only resulted in locomotor deficits comparable to those of rats with a complete spinal cord lesion (BBB score of 2). Also, Loy and coworkers [43,44] showed that a demyelinating lesion of the entire ventral white matter at the thoracic level abolished plantar stepping in rats (BBB score of about 6.5), while simultaneous demyelination of the ventrolateral funiculi and the dorsolateral funiculi evoked severe locomotor deficits, but plantar stepping was preserved.

It appears that plantar stepping after spinal cord injury is present if enough white matter is preserved within the region containing the dorsolateral and ventrolateral funiculi and the dorsal columns, where the vestibulo-, rubro- and reticulospinal, coeruleospinal, raphespinal and propriospinal tracts are located [43]. Hemisection of the spinal cord destroys all of the fibers going in the left half of the spinal cord, including those descending from the supraspinal structures, the propriospinal connections, as well as the ascending fibers from spinal structures lying below the injury. This lesion evokes immediate complete paralysis of the ipsilateral hindlimb which has been clearly demonstrated by Loy and Schucht [42–44]. But within several days (11 days after the spinal injury in our experiments) plantar stepping, as well as body weight support, recovers. The recovery of locomotor functions is an effect of changes taking place in the remaining fibers and neurons present in the right, intact part of the spinal cord. Recovery after spinal lateral hemisection was associated with an increased number of collaterals of reticulospinal fibers present in the half of the spinal cord contralateral to the lesion [4] and crossing the midline below the lesion, suggesting that sprouting of the spared contralateral reticulospinal fibers could be involved in recovery of locomotion. Collaterals of the spared reticulospinal fibers may also make contact with the propriospinal neurons, which double across the midline, once above the lesion and once below the lesion, acting on the neuronal network below the lesion as was shown by Filli and coworkers [45]. The loss of plantar walking ability, with respect to the ipisilateral hindlimb, is understandable, yet why is plantar walking in the contralateral hindlimb also impaired? In the acute stage, after spinal cord hemisection, this impairment might be due to trauma and spinal cord shock processes, inflammation and apoptosis, edema, glutamate excitotoxicity, the processes that transfer to the observed neurological deficits and also impairments in contralateral hindlimb functioning [46–48]. The chronic effect may be related to the damage of descending pathways, which act on the motoneurons and interneurons on the contralateral part of the spinal cord through commissural interneurons [49,50]. Another explanation may be the initial loss of serotonergic fibers on the right, contralateral to the lesion half of the spinal cord. The number of pixels one week after hemisection was 60% lower compared to the number of pixels counted in intact animals. To our knowledge, none of the other studies have shown a reduction in the number of descending fibers on the contralateral half of the spinal cord after hemisection.

In our experiments, some of the locomotor parameters returned to values obtained in intact animals, such as the fore-hindlimb coupling ratio, which reached control values around 18 days after hemisection. The ***r***-value describing the variability of intra- and interlimb coordination returned to the control values around 2 weeks after hemisection. However, the values of most measures of motor coordination were altered in hemisected rats indicating persistent locomotor disabilities. These parameters reached a plateau within 2 weeks or 1 month after hemisection. Parameters like the phase shift of EMG burst activities in inter- and intralimb coordination, the locomotor speed and the hindlimb abduction reached plateaus 2 weeks after hemisection, while the hindlimb base of support and relative foot print distance did so one month after hemisection. These results are partly consistent with results obtained in other studies. Most other studies have suggested that locomotion of rats after a lateral hemisection did not fully recover in both hindlimbs. Only in one study [9] did all of the hemisected rats recover normal locomotion after 2 weeks, and in another [40], locomotor movement fully recovered in the contralateral hindlimb. There is an agreement that locomotor parameters which did not return to the value obtained before hemisection reach a plateau, although precisely when this occurs is unclear and likely depends on experimental protocol. Several authors have shown that locomotion evaluated with BBB or Tarlov scales may reach a plateau 2 weeks [4,7,35], 2–6 weeks [40], 3 weeks [1,2] or 4 weeks [39] after hemisection. The other difference is the level of recovery. In several studies, rats reached a BBB score of 12, indicating that they recovered hindlimb plantar stepping and body weight support, but not fore-hindlimb coupling [1,7,35]. It was also revealed [8] that 21 days following spinal cord hemisection fore-hindlimb locomotor movements were uncoupled as measured by forelimb-hindlimb step ratio. In other studies (as well as in ours) fore-hindlimb coupling returned to normal [2,4,9,40].

### Recovery of coordination

One of the locomotor deficits observed within the first two weeks after hemisection was the presence of unequal rhythms of the fore- and hindlimb movements, which could be expressed as an unequal number of fore- and hindlimb steps counted in the same period of time, or as an unequal number of fore- and hindlimb EMG bursts in the flexor and extensor muscles. This observation is consistent with previous reports concerning locomotor behavior after partial spinal cord injuries in rats [8,51–53]. Here, we propose a method of quantifying this phenomenon by establishing the fore-hindlimb coupling ratio based on muscle EMG activity recordings.

After hemisection the mean coupling ratio for the ipsilateral pair of limbs was more severely reduced than for the contralateral ones, and within several days both CRs increased and did not differ from the value obtained in intact animals (11 and 14 days post hemisection for contralateral and ipsilateral pairs of limbs, respectively). Over the next few days, the CR value was close to 1, which suggested that different fore- hindlimb rhythms were occasionally present. The exact time point when restoration of equal rhythms occurs in the fore- and hindlimbs remains unclear. Górska and coworkers [53] suggested that after partial spinal cord injury in rats, this process depends on the extent of the lesion. For example, rats in which only the ventral funiculi were preserved showed recovery of equal rhythms of the fore- and hindlimbs 3.5 months after surgery. Garcia-Alias and colleagues [1] observed that 6 weeks after thoracic hemisection, only the rats that received treatment (Chondroitinase ABC or NR2D vectors or NT3-sectreting plugs or combined) were able to restore equal stride length of fore- and hindlimbs. The rats that did not receive any treatment still showed abnormal fore- hindlimb rhythms at this time-point (as indicated by the different stride lengths in the fore and hindlimbs). This result is contradictory to ours since at this time point, we did not observe any rhythmic differences between the fore- and hindlimbs.

Episodes of different rhythms have also been observed in cats. For example, [51] in cats with an over-hemisected spinal cord at the thoracic level, 2 weeks after injury, the duration of the step cycle of the hindlimbs was about 1000 ms and for the forelimbs it was 800 ms. Clearly, unbalanced rhythms of the fore- and hindlimb episodes were present. Also, Bem and collaborators [52] showed that in cats with subtotal spinal cord injury there is a difference in the duration of the step cycles between the fore- and hindlimbs.

Important elements in the mechanism of motor control responsible for producing and maintaining coordination of locomotor movements of both limb girdles are the long ascending and descending proprospinal pathways [54–56]. These pathways connect the cervical and lumbar enlargements, where the motoneurons of fore- and hindlimb muscles are located. The cells of these pathways project from the cervical to the lumbar segments and *vice versa*. For example, the proprospinal neurons of the cervical segment send projections to both halves of the spinal cord at the lumbar level [54]. It is believed that the proprospinal pathways are in the lateral columns, as damage of these columns induces the appearance of unequal rhythms in the fore- and hindlimbs in cats [52]. According to Górska and colleagues [53], the observed uncoupling might occur due to destruction of these fibers. The recovery of fore-hindlimb coupling observed in our experiments within 2–3 weeks after the lesion might result from increased length of the 5-HT fibers in the ipsilateral half of the spinal cord (about a 5-fold increase) as well as in the contralateral half of the spinal cord (about a 50% increase). It is also likely that the recovery of fore-hindlimb coordination depends on the connection between descending tracts from the supraspinal structures and the priopriospinal fibers, which was revealed in relation to the reticulospinal tract after lateral cervical hemisection [45].

Two other parameters characterizing the coordination of limb movements also changed after unilateral hemisection. After the lesion, the ***r***-value, which reflects the strength of coordination between the analyzed muscle burst onsets with respect to intra- and interlimb coordination dropped dramatically from about 0.9 to about 0.5, but after 2 weeks it returned to the value obtained in intact rats. The ***r***-value is a very sensitive tool for estimating the quality of locomotor movements, especially when they are severely deteriorated.

The timing and the pattern of muscle activity in locomotion is produced by the spinal network (CPG, Central Pattern Generator) and controlled by descending commands and sensory information from peripheral receptors. The weak correlation between muscle activities after the lesion might result from disorganization of the spinal network activity evoked by spinal shock. It is also likely that the weak descending influence on the excitability of motoneurons decreases the muscle force and evokes the loss of body weight support. During locomotion, the CPG receives misleading information from the hindlimbs that are being dragged with the dorsal surface of the foot contacting the ground, which may produce changes in the pattern of locomotion. Once the animals recover body weight support and plantar stepping, the ***r***-value returns to its value before the lesion, despite the fact that other locomotor parameters are still changed.

In intact animals, the phase shift of intralimb coordination (between l TA-Sol and r TA-Sol) was approximately 130±8°. Similar studies of intralimb coordination are rare, however [57], in cats, the phase shift of the antagonist muscles of the knee of the ipsilateral hindlimb, *vastus lateralis* (extensor) in relation to *semitendinosus* (flexor), was 0.35 before hemisection, which corresponds to 126°. This observation is consistent with our results. Moreover, Martinez and colleagues [57] did not observe any changes in this value 3 weeks after hemisection, which is also consistent with our findings.

Our results show that after hemisection the mean phase shift of interlimb coordination (between l-r TA and l-r Sol) decreased and no improvement was observed for the duration of the study. Barierre and coworkers [51] observed a similar phenomenon using a kinematic analysis of locomotion in cats after partial spinal cord injuries. These authors describe that in cats with a hemisection-like lesion, 20 days after the injury, the phase shift of the left-right hindlimb coupling decreased from 0.5 to a value of 0.4 (corresponding respectively to 180 and 144° on a 360° scale). Martinez and coworkers [57] also observed that, in cats, locomotion assessment after spinal cord hemisection showed that the right hip flexor *sartorius*, as well as the right knee extensor *vastus lateralis*, tended to contract earlier in the cycle of left *sartorius* and left *vastus lateralis*, respectively. They claim that after spinal cord hemisection the “cats probably developed behavioral strategies to compensate for the left hindlimb deficits by using the right hindlimb more.” Our results support this hypothesis.

### Recovery and subsequent decrement of spatial parameters of locomotor abilities after spinal cord hemisection—Catwalk results

In our study, before hemisection rats moved along the CatWalk runway with a mean speed of about 57 cm/s. The mean speed of intact rats tested in our experiments is within the range observed by the other authors [58,59] and our previous studies [23,53,60,61]. Two weeks after lesion, when the animals regained quadrupedal locomotion, the mean speed was about 38 cm/s, which is about 35% slower than before hemisection. In the following months, the mean speed of locomotion was similar, but 5 months after hemisection, it dropped again and was more than 50% slower compared to intact animals. Several studies have shown that after a severe spinal cord lesion, the speed of locomotion is less than before the injury [53,60,62]. The drop in speed may be due to a reduction in the propulsive forces or the loss of the body stability, because these lesions deprived the LH spinal motor centers of the supraspinal drive transmitted to the lumbar cord by the reticulospinal and vestibulospinal tracts [60]. A similar mechanism has been suggested [40] for alterations in ground reaction forces recorded in rats after ventrolateral spinal injury. But, to our knowledge, none of these previous studies described a secondary decrease in locomotion speed.

Our investigations showed that the BOS did not change in the forelimbs after hemisection, in contrast to the hindlimbs, in which the BOS increased by about 40% two weeks after hemisection. One month after hemisection the BOS decreased and was not different compared to pre-lesion values. Starting from the 2^nd^ month, the BOS increased gradually, and 3 and 5 month afterwards it was significantly greater than before hemisection.

An increase in hindlimb BOS is a characteristic phenomenon observed after a large spinal cord lesion or contusion [62]. BOS was used as a distinctive locomotor parameter after a lateral hemisection in many studies [1,2,6,36,63], and in most of these studies, the BOS increased starting from 2 to 10 weeks after the lesion, but a detailed time course of the changes in the BOS have not been analyzed. The initial increase in the BOS 2 weeks after the lesion was associated with very unstable hindlimb locomotor movements, resulting from decreased facilitation of the spinal network from the supraspinal structures and might indicate a strategy to deal with unstable body balance after SCI. In the weeks following, the BOS decreased, reaching the lowest value one month after hemisection at the peak of recovery and then gradually increased reaching the highest value 5 months after hemisection. A similar time course of these changes was seen for the left and right HA. This parameter was not analyzed by other authors, but we consider it potentially important for estimating the symmetry of the hind leg position during locomotion after asymmetrical lesions such as lateral hemisection. Interestingly, the abduction of the left and right hindlimb was similar at each time point after the lesion. Another parameter that changed after hemisection was the relative print distance (RPD). At moderate velocities intact rats usually place their hind foot close to the previous position of the ipsilateral fore foot. This probably makes positioning of the hindlimb, which is not performed under the visual control, more secure. In intact animals, the RPD is close to 0, but after hemisection it increased, since both of the hind feet were placed behind the fore feet. RPD reached a plateau one month after hemisection but 4 months later we observed an increase. Similar to hindlimb abduction, the RPD of the left limbs was not different from the RPD of right limbs. The above dissociation between fore- and hindlimb movements may result from weakened muscle force as a consequence of decreased facilitation from supraspinal structures associated with damaged descending spinal tracts and/or reduced connection between the fore-hindlimb spinal networks due to destroyed proprospinal neurons.

### Ipsi- and contralateral alterations of serotonergic innervation after spinal cord hemisection

Our analysis showed that after hemisection at the thoracic level, the length of the serotonergic fibers in the ventral horn of the lumbar spinal cord decreased. On the ipsilateral side, the decrease was substantial, and one week after lesion, the length of the serotonergic fibers did not exceed 1% of fiber length in the intact animals. In the following weeks their length increased reaching a plateau one month after the lesion, and over the next few months the fiber length stayed at a level of 5–6% of the total fiber length observed in the ventral horn of the spinal cords of intact animals. On the contralateral side, the decrease was less pronounced, and the length of the serotonergic fibers reduced to less than 50% of the fiber length measured in the spinal cords of the intact animals. However, it cannot be excluded that a transient decrease in the immunoreactivity on the contralateral side of the spinal cord was caused by a local decrease in 5-HT synthesis rather than fiber degeneration itself. In the following weeks the length of the fibers gradually increased, and 4 weeks after the lesion they were not different in comparison to intact animals. Three months after the hemisection, the mean serotonergic fiber length on the contralateral side was almost the same as in the intact animals, but 3 months later reductions in fiber length were observed.

The increase in serotonergic fiber density on the ipsilateral side we observed is lower than previous studies using rats [9,64] or mice [65,66]. In contrast, studies of reticulospinal projection after lateral thoracic hemisection show that there was no significant increase in the fiber density in the ventral horn at the lumbar spinal level [4]. However, it should be emphasized that, in many studies, the measures of fiber changes are mainly based on the immunoreactivity index or an optical density assessment. We found that this measurement gives only a general approximation of this process, rather than closely reflecting it. The determination of the nerve fiber density using optical density measurements has several disadvantages. For example, in the same image area, a smaller number of large diameter fibers can give the same density index as a larger number of fibers with a small diameter. The other is that immunofluorescence labeling is often non-uniform in a thick section (our case), and the image is often blurred [67]. Our observations indicate that the changes in serotonergic fiber patterns were very subtle, and therefore we decided to perform further analysis using a skeletonization algorithm. This method is used for investigating the length of longitudinal biostructures like blood vessels [68]. The initial reduction in serotonergic fiber length on the contralateral side of the spinal cord has not been described previously. Moreover, studies investigating the plasticity of other descending fibers after lateral hemisection did not show any changes on the contralateral side of the spinal cord [4,9].

Our results show that after lateral hemisection at the thoracic level, an increase in the length of serotonergic fibers in the ipsilateral lumbar (6%) was identified 6 months after hemisection. Despite the initial decrease in serotonergic fiber length in the ventral horn to about 40% of that in the intact animals on the contralateral side of the spinal cord, 4 weeks after hemisection, fiber length almost returned control values. However, it should be considered that in the intact animals serotonergic fibers are overlapping and their length can be underestimated. Therefore, the stain of the intact tissue (see Fig 5A3) appears more intense than in the ventral horn 3 months after the hemisection (see Fig 5D3) despite the similar number of pixels in the skeletonized image.

### Serotonergic fiber regrowth and the recovery of locomotion

The pronounced role of serotonin, its receptor agonists or antagonists, as well as serotonin transplants in the recovery of locomotion after spinal cord injuries is well established [16,17,20]. However, it is unclear how the process of serotonin fiber restoration proceeds after partial spinal cord injury and the contribution of this process to locomotor function recovery is unknown. Saruhashi and coworkers [9] showed a correlation between changes in the density of serotonergic fibers or the 5-HT immunoreactive terminals in the lumbar spinal cord and locomotor recovery.

Our analysis showed a correlation between the length of serotonergic fibers in the ipsilateral and contralateral ventral horns and the *r*-values that describe the strength of the coupling between the burst onsets of EMG in a given pair of muscles, either with respect to intra- or interlimb coordination.

Serotonin exerts very complex effects on processes related to locomotion generation, such as increments of excitability of motoneurons or increased activity of the central pattern generator [13,28,69–71]. Upon application onto the isolated neonatal spinal rat cord, serotonin induces the generation of alternating, rhythmic discharges in the ventral roots (fictive locomotion) [69,72]. Therefore, it is probable that an increased serotonin influx *via* the regrowth of fibers caused the improvement in locomotion regularity as measured by the increased ***r***-value.

Our observation of the deterioration of locomotor hindlimb performance in the chronic stage was related to several locomotor parameters: locomotor speed; intralimb coordination; hindlimb base of support (along with this parameter also hindlimb abduction) and relative foot print position. This deterioration was not significantly correlated with a decrease in spinal cord serotonergic fiber length, although we measured this parameter only within a selected sample of the ventral horn. There might be other sites of termination that could contribute to the deterioration. Moreover, it is worth pointing out some coincidences between changes in locomotor performance (outcome) and serotonergic fibers length. The recovery of several locomotor parameters (locomotor velocity, print distance, base of support etc.) within the first 4 weeks after the lesion occurred concomitantly with the increase in the length of serotonergic fibers; from 1% to more than 5% on the ipsilateral side and from about 40% to almost 100% of the fiber length in intact animals. Also, a late deterioration of these locomotor parameters was associated with a late decrease in serotonergic fiber length on the contralateral side. We believe that changes in locomotor performance after thoracic hemisection are not related exclusively to changes in the density of serotonergic fibers and that many other descending tracts are involved in the process of recovery and the late deterioration of locomotion.

This deterioration might be due to progressive changes in the physiology of spared fibers on the contralateral side of the spinal cord. Arvanian and coworkers [7] suggested that chronic hemisection induces a pathological state in the physiology of spared spinal fibers that starts about 1–2 weeks after hemisection. They observed that the transmission through the surviving axons was delayed. Moreover, in the subsequent weeks (up to week 14), the magnitude of the lumbar motoneuron responses to thoracic- (above the lesion) derived electrical stimuli (either on the ipsilateral or contralateral side) was reduced. Another possible mechanism is the accumulation of chondroitin sulfate proteoglycans in the neighborhood of the spared axons, which could reduce their ability to conduct impulses [2,73].

In conclusion, our findings suggest that after lateral hemisection rats recover substantial locomotor ability with body weight support and plantar stepping with toe clearance about 2–4 weeks after the lesion, but several locomotor parameters did not return to pre-lesion values. Moreover, we showed for the first time that a late deterioration of locomotion occurs 4–5 months after hemisection. We associated the temporal changes in locomotor function with the length of serotonergic fibers not only on the ipsilateral but also on the side contralateral to hemisection. Of all the locomotor measures used in our study, we only found a correlation between serotonergic fiber length and limb coordination. However, we have to consider that many other descending systems are involved in the changes in locomotion that occur after spinal cord injury. More studies are warranted to test this hypothesis, for instance, locally blocking not only 5-HT receptors but also receptors involved in other descending systems at the lumbar spinal level to evaluate their importance in locomotion after spinal injury (for the method describing intrathecal drug administration see [17,74]).

## Supporting Information

S1 Table The relation between fore- and hindlimb rhythms during locomotion (EMG analysis). The table contains means of the fore-hindlimb coupling ratio (CR) defined as: the ratio of the number of EMG bursts of a forelimb muscle to the number of EMG bursts of a selected hindlimb muscle within a run, containing 10–15 forelimb cycles in individual rats, and the means±SEM calculated in the various groups of animals for particular time points. Abbreviations: dpo- days post spinal cord hemisection.
(DOCX)

S2 Table The strength of inter- and intralimb coordination (EMG analysis). The table contains means of ***r***-value of intra- and interlimb established in Polar Plot analysis for individual rats and the means±SEM calculated in the various groups of animals for particular time points up to 4 weeks. Abbreviations: **l-r TA**—interlimb coordination established based on left—right TA EMG burst activity; **l-r Sol—**interlimb coordination established based on left—right Sol EMG burst activity; **l Sol-l TA; r Sol-r TA—**intralimb coordination established based on Sol versus TA in both hindlimbs separately.
(DOCX)

S3 Table The phase shift of inter- and intralimb coordination (EMG analysis). The table contains means of phase shifts between onsets of EMG bursts in specific muscles for intra- and interlimb coordination in individual rats and the means±SEM calculated in the various groups of animals for particular time points. Abbreviations: **l-r TA**—interlimb coordination established based on left—right TA EMG burst activity; **l-r Sol—**interlimb coordination established based on left—right Sol EMG burst activity; **l Sol-l TA; r Sol-r TA—**intralimb coordination established based on Sol versus TA in both hindlimbs separately.
(DOCX)

S4 Table Results of CatWalk analysis showing the Base of Support (BOS). The table contains means of hindlimb BOS in individual rats and the means±SEM calculated in the various groups of animals for particular time points.
(DOCX)

S5 Table Results of CatWalk analysis showing the abduction of hindlimbs (HA). The table contains means of HA for left and right hindlimbs in individual rats and the means±SEM calculated in the various groups of animals for particular time points. Abbreviations: **RF**- right forelimb; **RH–**right hindlimb; **LF-** left forelimb; **LH–**left hindlimb.
(DOCX)

S6 Table Results of CatWalk analysis showing the Relative Print Distance (RPD). The table contains means of RPD between left and right fore—hindlimb pairs in individual rats and the means±SEM calculated in the various groups of animals for particular time points.
Abbreviations: **RH–**right hindlimb; **LH–**left hindlimb; wpo- weeks; mpo- months post spinal cord hemisection.
(DOCX)

S7 Table Results of CatWalk analysis showing the speed of locomotion (V). The table contains means of V in individual rats and the means±SEM calculated in the various groups of animals for particular time points. Abbreviations: wpo- weeks; mpo- months post spinal cord hemisection.
(DOCX)

S8 Table The length of serotonergic fibers on ipsilateral and contralateral sides of the spinal cord. The table contains means of pixels established for individual rats in the left and right ventral horns of their spinal cords and the means±SEM in the various groups of animals for particular time points. Abbreviations: wpo- weeks; mpo- months post spinal cord hemisection.
(DOCX)

S9 Table Correlation between ipsilateral or contralateral serotonergic fiber lengths and locomotor parameters. The table contains coefficient of correlation and its significance for the relationships between various locomotor parameters and the serotonergic fiber lengths in the left and right ventral horns of their spinal cords. Abbreviations: ***r***-value—the strength of intra- or interlimb coordination, CC—correlation coefficient, *p*–significance of the CC
(DOCX)

## References

[1] Garcia-Alias G, Petrosyan HA, Schnell L, Horner PJ, Bowers WJ, Mendell LM, et al Chondroitinase ABC combined with neurotrophin NT-3 secretion and NR2D expression promotes axonal plasticity and functional recovery in rats with lateral hemisection of the spinal cord. J Neurosci. 2011; 31: 17788–17799. 10.1523/JNEUROSCI.4308-11.2011 22159095 PMC3758578

[2] Petrosyan HA, Hunanyan AS, Alessi V, Schnell L, Levine J, Arvanian VL. Neutralization of inhibitory molecule NG2 improves synaptic transmission, retrograde transport, and locomotor function after spinal cord injury in adult rats. J Neurosci. 2013; 33: 4032–4043. 10.1523/JNEUROSCI.4702-12.2013 23447612 PMC6619302

[3] Schnell L, Hunanyan AS, Bowers WJ, Horner PJ, Federoff HJ, Gullo M, et al Combined delivery of Nogo-A antibody, neurotrophin-3 and the NMDA-NR2d subunit establishes a functional 'detour' in the hemisected spinal cord. Eur J Neurosci. 2011; 34: 1256–1267. 10.1111/j.1460-9568.2011.07862.x 21995852 PMC3195885

[4] Ballermann M, Fouad K. Spontaneous locomotor recovery in spinal cord injured rats is accompanied by anatomical plasticity of reticulospinal fibers. Eur J Neurosci. 2006; 23: 1988–1996. 16630047 10.1111/j.1460-9568.2006.04726.x

[5] Basso DM, Beattie MS, Bresnahan JC. Graded histological and locomotor outcomes after spinal cord contusion using the NYU weight-drop device versus transection. Exp Neurol. 1996; 139: 244–256. 8654527 10.1006/exnr.1996.0098

[6] Redondo-Castro E, Torres-Espin A, Garcia-Alias G, Navarro X. Quantitative assessment of locomotion and interlimb coordination in rats after different spinal cord injuries. J Neurosci Methods. 2013; 213: 165–178. 10.1016/j.jneumeth.2012.12.024 23291085

[7] Arvanian VL, Schnell L, Lou L, Golshani R, Hunanyan A, Ghosh A, et al Chronic spinal hemisection in rats induces a progressive decline in transmission in uninjured fibers to motoneurons. Exp Neurol. 2009; 216: 471–480. 10.1016/j.expneurol.2009.01.004 19320005 PMC2889190

[8] Shah PK, Garcia-Alias G, Choe J, Gad P, Gerasimenko Y, Tillakaratne N, et al Use of quadrupedal step training to re-engage spinal interneuronal networks and improve locomotor function after spinal cord injury. Brain. 2013; 136: 3362–3377. 10.1093/brain/awt265 24103912 PMC3808689

[9] Saruhashi Y, Young W, Perkins R. The recovery of 5-HT immunoreactivity in lumbosacral spinal cord and locomotor function after thoracic hemisection. Exp Neurol. 1996; 139: 203–213. 8654523 10.1006/exnr.1996.0094

[10] Rossignol S, Barriere G, Alluin O, Frigon A. Re-expression of locomotor function after partial spinal cord injury. Physiology (Bethesda). 2009; 24: 127–139. 19364915 10.1152/physiol.00042.2008

[11] Dietz V. Behavior of spinal neurons deprived of supraspinal input. Nat Rev Neurol. 2010; 6: 167–174. 10.1038/nrneurol.2009.227 20101254

[12] Fung SJ, Barnes CD. Raphe-produced excitation of spinal cord motoneurons in the cat. Neurosci Lett. 1989; 103: 185–190. 2549471 10.1016/0304-3940(89)90573-9

[13] Liu J, Jordan LM. Stimulation of the parapyramidal region of the neonatal rat brain stem produces locomotor-like activity involving spinal 5-HT7 and 5-HT2A receptors. J Neurophysiol. 2005; 94: 1392–1404. 15872068 10.1152/jn.00136.2005

[14] Sławińska U, Miazga K, Jordan LM. The role of serotonin in the control of locomotor movements and strategies for restoring locomotion after spinal cord injury. Acta Neurobiol Exp (Wars). 2014; 74: 172–187. 24993627 10.55782/ane-2014-1983

[15] Wang MY, Dun NJ. 5-Hydroxytryptamine responses in neonate rat motoneurones in vitro. J Physiol. 1990; 430: 87–103. 2150862 10.1113/jphysiol.1990.sp018283 PMC1181729

[16] Feraboli-Lohnherr D, Orsal D, Yakovleff A, Gimenez y Ribotta M, Privat A. Recovery of locomotor activity in the adult chronic spinal rat after sublesional transplantation of embryonic nervous cells: specific role of serotonergic neurons. Exp Brain Res. 1997; 113: 443–454. 9108211 10.1007/pl00005597

[17] Majczyński H, Maleszak K, Cabaj A, Sławińska U. Serotonin-related enhancement of recovery of hind limb motor functions in spinal rats after grafting of embryonic raphe nuclei. J Neurotrauma. 2005; 22: 590–604. 15892603 10.1089/neu.2005.22.590

[18] Sławińska U, Majczyński H, Djavadian R. Recovery of hindlimb motor functions after spinal cord transection is enhanced by grafts of the embryonic raphe nuclei. Exp Brain Res. 2000; 132: 27–38. 10836633 10.1007/s002219900323

[19] Sławińska U, Miazga K, Cabaj AM, Leszczyńska AN, Majczyński H, Nagy JI, et al Grafting of fetal brainstem 5-HT neurons into the sublesional spinal cord of paraplegic rats restores coordinated hindlimb locomotion. Exp Neurol. 2013; 247: 572–581. 10.1016/j.expneurol.2013.02.008 23481546

[20] Gimenez y Ribotta M, Orsal D, Feraboli-Lohnherr D, Privat A. Recovery of locomotion following transplantation of monoaminergic neurons in the spinal cord of paraplegic rats. Ann N Y Acad Sci. 1998; 860: 393–411. 9928327 10.1111/j.1749-6632.1998.tb09064.x

[21] Ribotta MG, Provencher J, Feraboli-Lohnherr D, Rossignol S, Privat A, et al Activation of locomotion in adult chronic spinal rats is achieved by transplantation of embryonic raphe cells reinnervating a precise lumbar level. J Neurosci. 2000; 20: 5144–5152. 10864971 10.1523/JNEUROSCI.20-13-05144.2000 PMC6772289

[22] Gimenez y Ribotta M, Rajaofetra N, Morin-Richaud C, Alonso G, Bochelen D, Sandillon F, et al Oxysterol (7 beta-hydroxycholesteryl-3-oleate) promotes serotonergic reinnervation in the lesioned rat spinal cord by reducing glial reaction. J Neurosci Res. 1995; 41: 79–95. 7674380 10.1002/jnr.490410110

[23] Majczyński H, Maleszak K, Górska T, Sławińska U. Comparison of two methods for quantitative assessment of unrestrained locomotion in the rat. J Neurosci Methods. 2007; 163: 197–207. 17418901 10.1016/j.jneumeth.2007.02.023

[24] Batschelet E. Circukar Statistics in Biology. New York: Academic Press 1981.

[25] Cowley KC, Zaporozhets E, Maclean JN, Schmidt BJ. Is NMDA receptor activation essential for the production of locomotor-like activity in the neonatal rat spinal cord? J Neurophysiol. 2005; 94: 3805–3814. 16120672 10.1152/jn.00016.2005

[26] Kjaerulff O, Kiehn O. Distribution of networks generating and coordinating locomotor activity in the neonatal rat spinal cord in vitro: a lesion study. J Neurosci. 1996; 16: 5777–5794. 8795632 10.1523/JNEUROSCI.16-18-05777.1996 PMC6578971

[27] Zar JH. Circular distribution In: McElroy WD, Swanson C.P., editor. Biostatistical Analysis. Englewood Cliffs: Prentice Hall 1974 pp. 310–327.

[28] Sławińska U, Majczyński H, Dai Y, Jordan LM. The upright posture improves plantar stepping and alters responses to serotonergic drugs in spinal rats. J Physiol. 2012; 590: 1721–1736. 10.1113/jphysiol.2011.224931 22351637 PMC3413485

[29] Jordan LM, Sławińska U. Chapter 12—modulation of rhythmic movement: control of coordination. Prog Brain Res. 2011; 188: 181–195. 10.1016/B978-0-444-53825-3.00017-6 21333810

[30] Liu J, Akay T, Hedlund PB, Pearson KG, Jordan LM. Spinal 5-HT7 receptors are critical for alternating activity during locomotion: in vitro neonatal and in vivo adult studies using 5-HT7 receptor knockout mice. J Neurophysiol. 2009; 102: 337–348. 10.1152/jn.91239.2008 19458153

[31] Madriaga MA, McPhee LC, Chersa T, Christie KJ, Whelan PJ. Modulation of locomotor activity by multiple 5-HT and dopaminergic receptor subtypes in the neonatal mouse spinal cord. J Neurophysiol. 2004; 92: 1566–1576. 15163678 10.1152/jn.01181.2003

[32] Pearlstein E, Ben Mabrouk F, Pflieger JF, Vinay L. Serotonin refines the locomotor-related alternations in the in vitro neonatal rat spinal cord. Eur J Neurosci. 2005; 21: 1338–1346. 15813943 10.1111/j.1460-9568.2005.03971.x

[33] Sławińska U, Miazga K, Jordan LM. 5-HT(2) and 5-HT(7) receptor agonists facilitate plantar stepping in chronic spinal rats through actions on different populations of spinal neurons. Front Neural Circuits. 2014; 8: 95 10.3389/fncir.2014.00095 25191231 PMC4137449

[34] Dietz V. Neuronal plasticity after a human spinal cord injury: positive and negative effects. Exp Neurol. 2012; 235: 110–115. 10.1016/j.expneurol.2011.04.007 21530507

[35] Cloud BA, Ball BG, Chen BK, Knight AM, Hakim JS, Ortiz AM, et al Hemisection spinal cord injury in rat: the value of intraoperative somatosensory evoked potential monitoring. J Neurosci Methods. 2012; 211: 179–184. 10.1016/j.jneumeth.2012.08.024 22960163 PMC3491113

[36] Deumens R, Van Gorp SF, Bozkurt A, Beckmann C, Fuhrmann T, Montzka K, et al Motor outcome and allodynia are largely unaffected by novel olfactory ensheathing cell grafts to repair low-thoracic lesion gaps in the adult rat spinal cord. Behav Brain Res. 2013; 237: 185–189. 10.1016/j.bbr.2012.09.036 23022748

[37] Gulino R, Dimartino M, Casabona A, Lombardo SA, Perciavalle V Synaptic plasticity modulates the spontaneous recovery of locomotion after spinal cord hemisection. Neurosci Res. 2007; 57: 148–156. 17083989 10.1016/j.neures.2006.10.001

[38] Hains BC, Johnson KM, McAdoo DJ, Eaton MJ, Hulsebosch CE. Engraftment of serotonergic precursors enhances locomotor function and attenuates chronic central pain behavior following spinal hemisection injury in the rat. Exp Neurol. 2001; 171: 361–378. 11573989 10.1006/exnr.2001.7751

[39] Voda J, Yamaji T, Gold BG. Neuroimmunophilin ligands improve functional recovery and increase axonal growth after spinal cord hemisection in rats. J Neurotrauma. 2005; 22: 1150–1161. 16238491 10.1089/neu.2005.22.1150

[40] Webb AA, Muir GD. Compensatory locomotor adjustments of rats with cervical or thoracic spinal cord hemisections. J Neurotrauma. 2002; 19: 239–256. 11893025 10.1089/08977150252806983

[41] Wong JK, Steward O. One day of motor training with amphetamine impairs motor recovery following spinal cord injury. Exp Neurol. 2012; 233: 693–707. 10.1016/j.expneurol.2011.08.011 22078754

[42] Schucht P, Raineteau O, Schwab ME, Fouad K. Anatomical correlates of locomotor recovery following dorsal and ventral lesions of the rat spinal cord. Exp Neurol. 2002; 176: 143–153. 12093091 10.1006/exnr.2002.7909

[43] Loy DN, Talbott JF, Onifer SM, Mills MD, Burke DA, Dennison JB, et al Both dorsal and ventral spinal cord pathways contribute to overground locomotion in the adult rat. Exp Neurol. 2002; 177: 575–580. 12429203 10.1006/exnr.2002.7959

[44] Loy DN, Magnuson DS, Zhang YP, Onifer SM, Mills MD, Cao QL, et al Functional redundancy of ventral spinal locomotor pathways. J Neurosci. 2002; 22: 315–323. 11756515 10.1523/JNEUROSCI.22-01-00315.2002 PMC6757623

[45] Filli L, Engmann AK, Zorner B, Weinmann O, Moraitis T, Gullo M, et al Bridging the gap: a reticulo-propriospinal detour bypassing an incomplete spinal cord injury. J Neurosci. 2014; 34: 13399–13410. 10.1523/JNEUROSCI.0701-14.2014 25274818 PMC6608315

[46] Borgens RB, Liu-Snyder P. Understanding secondary injury. Q Rev Biol. 2012; 87: 89–127. 22696939 10.1086/665457

[47] Guest JD, Hiester ED, Bunge RP. Demyelination and Schwann cell responses adjacent to injury epicenter cavities following chronic human spinal cord injury. Exp Neurol. 2005; 192: 384–393. 15755556 10.1016/j.expneurol.2004.11.033

[48] Zhang N, Yin Y, Xu SJ, Wu YP, Chen WS. Inflammation & apoptosis in spinal cord injury. Indian J Med Res. 2012; 135: 287–296. 22561613 PMC3361863

[49] Bannatyne BA, Edgley SA, Hammar I, Jankowska E, Maxwell DJ. Networks of inhibitory and excitatory commissural interneurons mediating crossed reticulospinal actions. Eur J Neurosci. 2003; 18: 2273–2284. 14622188 10.1046/j.l460-9568.2003.02973.x PMC1971243

[50] Hammar I, Bannatyne BA, Maxwell DJ, Edgley SA, Jankowska E. The actions of monoamines and distribution of noradrenergic and serotoninergic contacts on different subpopulations of commissural interneurons in the cat spinal cord. Eur J Neurosci. 2004; 19: 1305–1316. 15016088 10.1111/j.l460-9568.2004.03239.x PMC1971244

[51] Barriere G, Frigon A, Leblond H, Provencher J, Rossignol S. Dual spinal lesion paradigm in the cat: evolution of the kinematic locomotor pattern. J Neurophysiol. 2010; 104: 1119–1133. 10.1152/jn.00255.2010 20573971

[52] Bem T, Gorska T, Majczynski H, Zmyslowski W. Different patterns of fore-hindlimb coordination during overground locomotion in cats with ventral and lateral spinal lesions. Exp Brain Res. 1995; 104: 70–80. 7621942 10.1007/BF00229856

[53] Górska T, Chojnicka-Gittins B, Majczyński H, Zmysłowski W. Changes in forelimb-hindlimb coordination after partial spinal lesions of different extent in the rat. Behav Brain Res. 2013; 239: 121–138. 10.1016/j.bbr.2012.10.054 23142611

[54] Brockett EG, Seenan PG, Bannatyne BA, Maxwell DJ. Ascending and descending propriospinal pathways between lumbar and cervical segments in the rat: evidence for a substantial ascending excitatory pathway. Neuroscience. 2013; 240: 83–97. 10.1016/j.neuroscience.2013.02.039 23454541

[55] Juvin L, Simmers J, Morin D. Propriospinal circuitry underlying interlimb coordination in mammalian quadrupedal locomotion. J Neurosci. 2005; 25: 6025–6035. 15976092 10.1523/JNEUROSCI.0696-05.2005 PMC6724791

[56] Krutki P. Bilateral projection of neurones of the C6 segment to S1 and S2 segments of the spinal cord in the cat. Acta Neurobiol Exp (Wars). 1997; 57: 1–9. 10.55782/ane-1997-1205 9407686

[57] Martinez M, Delivet-Mongrain H, Leblond H, Rossignol S. Recovery of hindlimb locomotion after incomplete spinal cord injury in the cat involves spontaneous compensatory changes within the spinal locomotor circuitry. J Neurophysiol. 2011; 106: 1969–1984. 10.1152/jn.00368.2011 21775717

[58] Hruska RE, Silbergeld EK. Abnormal locomotion in rats after bilateral intrastriatal injection of kainic acid. Life Sci. 1979; 25: 181–193. 491844 10.1016/0024-3205(79)90390-4

[59] Koopmans GC, Deumens R, Brook G, Gerver J, Honig WM, Hamers FP, et al Strain and locomotor speed affect over-ground locomotion in intact rats. Physiol Behav. 2007; 92: 993–1001. 17959205 10.1016/j.physbeh.2007.07.018

[60] Górska T, Chojnicka-Gittins B, Majczyński H, Zmysłowski W. Overground locomotion after incomplete spinal lesions in the rat: quantitative gait analysis. J Neurotrauma. 2007; 24: 1198–1218. 17610359 10.1089/neu.2006.0219

[61] Górska T, Chojnicka-Gittins B, Majczyński H, Zmysłowski W. Recovery of overground locomotion following partial spinal lesions of different extent in the rat. Behav Brain Res. 2009; 196: 286–296. 10.1016/j.bbr.2008.09.019 18940200

[62] Hamers FP, Lankhorst AJ, van Laar TJ, Veldhuis WB, Gispen WH. Automated quantitative gait analysis during overground locomotion in the rat: its application to spinal cord contusion and transection injuries. J Neurotrauma. 2001; 18: 187–201. 11229711 10.1089/08977150150502613

[63] Hunanyan AS, Alessi V, Patel S, Pearse DD, Matthews G, Arvanian VL. Alterations of action potentials and the localization of Nav1.6 sodium channels in spared axons after hemisection injury of the spinal cord in adult rats. J Neurophysiol. 2011; 105: 1033–1044. 10.1152/jn.00810.2010 21177993 PMC3074424

[64] Saruhashi Y, Matsusue Y, Fujimiya M. The recovery of 5-HT transporter and 5-HT immunoreactivity in injured rat spinal cord. Arch Orthop Trauma Surg. 2009; 129: 1279–1285. 10.1007/s00402-008-0754-z 18825396

[65] Camand E, Morel MP, Faissner A, Sotelo C, Dusart I. Long-term changes in the molecular composition of the glial scar and progressive increase of serotoninergic fibre sprouting after hemisection of the mouse spinal cord. Eur J Neurosci. 2004; 20: 1161–1176. 15341588 10.1111/j.1460-9568.2004.03558.x

[66] Menet V, Prieto M, Privat A, Gimenez y Ribotta M. Axonal plasticity and functional recovery after spinal cord injury in mice deficient in both glial fibrillary acidic protein and vimentin genes. Proc Natl Acad Sci USA. 2003; 100: 8999–9004. 12861073 10.1073/pnas.1533187100 PMC166427

[67] Sathyanesan A, Ogura T, Lin W. Automated measurement of nerve fiber density using line intensity scan analysis. J Neurosci Methods. 2012; 206: 165–175. 10.1016/j.jneumeth.2012.02.019 22613744 PMC3358701

[68] Yim PJ, Choyke PL, Summers RM. Gray-scale skeletonization of small vessels in magnetic resonance angiography. IEEE Trans Med Imaging. 2000; 19: 568–576. 11026460 10.1109/42.870662

[69] Cowley KC, Schmidt BJ. Regional distribution of the locomotor pattern-generating network in the neonatal rat spinal cord. J Neurophysiol. 1997; 77: 247–259. 9120567 10.1152/jn.1997.77.1.247

[70] Noga BR, Johnson DM, Riesgo MI, Pinzon A. Locomotor-activated neurons of the cat. I. Serotonergic innervation and co-localization of 5-HT7, 5-HT2A, and 5-HT1A receptors in the thoraco-lumbar spinal cord. J Neurophysiol. 2009; 102: 1560–1576. 10.1152/jn.91179.2008 19571190 PMC2746795

[71] Takakusaki K, Kohyama J, Matsuyama K, Mori S. Synaptic mechanisms acting on lumbar motoneurons during postural augmentation induced by serotonin injection into the rostral pontine reticular formation in decerebrate cats. Exp Brain Res. 1993; 93: 471–482. 8519336 10.1007/BF00229362

[72] Kiehn O, Kjaerulff O. Spatiotemporal characteristics of 5-HT and dopamine-induced rhythmic hindlimb activity in the in vitro neonatal rat. J Neurophysiol. 1996; 75: 1472–1482. 8727391 10.1152/jn.1996.75.4.1472

[73] Hunanyan AS, Garcia-Alias G, Alessi V, Levine JM, Fawcett JW, Mendell LM, et al Role of chondroitin sulfate proteoglycans in axonal conduction in Mammalian spinal cord. J Neurosci. 2010; 30: 7761–7769. 10.1523/JNEUROSCI.4659-09.2010 20534825 PMC3531897

[74] Antri M, Mouffle C, Orsal D, Barthe JY. 5-HT1A receptors are involved in short- and long-term processes responsible for 5-HT-induced locomotor function recovery in chronic spinal rat. Eur J Neurosci. 2003; 18: 1963–1972. 14622228 10.1046/j.1460-9568.2003.02916.x

