# Supplementary material for: Thoracic Hemisection in Rats Results in Initial Recovery Followed by a Late Decrement in Locomotor Movements, with Changes in Coordination Correlated with Serotonergic Innervation of the Ventral Horn
Source: PLoS One. 2015 Nov 25;10(11):e0143602. doi: 10.1371/journal.pone.0143602 (PMC4659566; doi:10.1371/journal.pone.0143602)
Supplement: S1 Table — The table contains means of the fore-hindlimb coupling ratio (CR) defined as: the ratio of the number of EMG bursts of a forelimb muscle to the number of EMG bursts of a selected hindlimb muscle within a run, containing 10–15 forelimb cycles in individual rats, and the means±SEM calculated in the various groups of animals for particular time points. Abbreviations: dpo- days post spinal cord hemisection. (DOCX) [file pone.0143602.s001.docx]

**S1 Table. The relation between fore- and hindlimb rhythms during locomotion (EMG analysis).**

| **l TA-l Bic** | Intact | 5dpo | 7dpo | 11dpo | 14dpo | 18dpo | 21dpo | 28dpo | 32dpo |
| --- | --- | --- | --- | --- | --- | --- | --- | --- | --- |
| 1 | 0.982759 | 0.019231 | 0.645833 | 0.836364 | 0.76 | 0.942308 | 0.90625 | 0.836735 | 0.978261 |
| 2 | 0.989825 | 0.116279 | 0.538462 | 0.92 | 0.878049 | 0.977273 | 1 | 0.8125 | 0.734694 |
| 3 | 0.979167 | 0.177215 | 0.788732 | 0.851064 | 0.745098 | 0.676056 | 0.981982 | 1 | 0.954545 |
| 4 | 0.93871 | 0.086957 | 0.826087 | 0.851064 | 0.840909 | 0.959184 | 1.032258 | 1 | 0.972603 |
| 5 | 0.969231 | 0.078431 | 0.533333 | 0.765306 | 0.553571 | 1 |  | 1 | 1 |
| 6 | 1 | 0.434783 | 0.067797 | 0.963415 | 0.982456 | 0.961538 |  | 1 |  |
| 7 | 1 |  | 0.823529 |  | 0.95122 |  |  | 0.980198 |  |
| 8 | 0.925 |  | 0.86 |  | 0.898305 |  |  | 1 |  |
| 9 | 0.964286 |  | 0.5 |  |  |  |  |  |  |
| 10 | 1 |  | 0.050847 |  |  |  |  |  |  |
| 11 | 0.985714 |  | 0.781818 |  |  |  |  |  |  |
| 12 |  |  | 0.629032 |  |  |  |  |  |  |
|  |  |  |  |  |  |  |  |  |  |
| mean | 0.975881 | 0.152149 | 0.587123 | 0.864535 | 0.826201 | 0.919393 | 0.980123 | 0.953679 | 0.928021 |
| SEM | 0.007534 | 0.060318 | 0.083638 | 0.0282 | 0.048814 | 0.04931 | 0.02673 | 0.02836 | 0.048873 |

| **r TA-r Bic** | Intact | 5dpo | 7dpo | 11dpo | 14dpo | 18dpo | 21dpo | 28dpo | 32dpo |
| --- | --- | --- | --- | --- | --- | --- | --- | --- | --- |
| 1 | 1 | 0.384615 | 0.604167 | 0.727273 | 0.7 | 0.807692 | 0.71875 | 0.673469 | 1 |
| 2 | 0.982281 | 0.465116 | 0.423077 | 0.62 | 0.902439 | 0.977273 | 1 | 0.729167 | 0.77551 |
| 3 | 1 | 0.417722 | 0.56338 | 0.829787 | 0.823529 | 0.760563 | 0.981982 | 0.977273 | 1 |
| 4 | 0.973118 | 0.586957 | 0.681159 | 0.637931 | 1 | 0.959184 | 1 | 1 | 0.958904 |
| 5 | 1 | 0.54902 | 0.666667 | 0.887755 | 0.75 | 1 |  | 0.981818 | 1 |
| 6 | 1 | 0.565217 | 0.288136 | 1 | 1 | 0.980769 |  | 1 |  |
| 7 | 1 |  | 0.838235 |  | 0.97561 |  |  | 0.980198 |  |
| 8 | 1 |  | 0.8 |  | 1 |  |  | 1 |  |
| 9 | 1 |  | 0.931034 |  |  |  |  |  |  |
| 10 | 0.987179 |  | 0.932203 |  |  |  |  |  |  |
| 11 | 0.985714 |  | 0.672727 |  |  |  |  |  |  |
| 12 |  |  | 0.516129 |  |  |  |  |  |  |
|  |  |  |  |  |  |  |  |  |  |
| mean | 0.993481 | 0.494774 | 0.659743 | 0.783791 | 0.893947 | 0.914247 | 0.925183 | 0.917741 | 0.946883 |
| SEM | 0.00292 | 0.034329 | 0.058069 | 0.060819 | 0.043048 | 0.04193 | 0.068942 | 0.047637 | 0.043576 |

| **l Sol-l Tri** | Intact | 5dpo | 7dpo | 11dpo | 14dpo | 18dpo | 21dpo | 28dpo | 32dpo |
| --- | --- | --- | --- | --- | --- | --- | --- | --- | --- |
| 1 | 1 | 0.019231 | 0.229167 | 1 | 0.62 | 0.846154 | 0.84375 | 0.734694 | 0.978261 |
| 2 | 0.973684 | 0.046512 | 0.173077 | 0.82 | 1 | 0.954545 | 1 | 0.895833 | 0.795918 |
| 3 | 1 | 0.189873 | 0.591549 | 0.957447 | 0.784314 | 0.71831 | 1 | 0.977273 | 1 |
| 4 | 0.994624 | 0.108696 | 0.028986 | 0.344828 | 0.795455 | 0.959184 | 1 | 1 | 0.958904 |
| 5 | 0.953846 | 0.156863 | 0.55 | 0.77551 | 0.767857 | 1 |  | 0.981818 | 0.987342 |
| 6 | 1 | 0.23913 | 0.220339 | 0.987805 | 1 | 0.961538 |  | 1 |  |
| 7 | 0.982143 |  | 0.823529 |  | 0.95122 |  |  | 0.970297 |  |
| 8 | 1 |  | 0.64 |  | 0.932203 |  |  | 1 |  |
| 9 | 0.982143 |  | 0.189655 |  |  |  |  |  |  |
| 10 | 0.974359 |  | 0.050847 |  |  |  |  |  |  |
| 11 | 1 |  | 0.836364 |  |  |  |  |  |  |
| 12 |  |  | 0.741935 |  |  |  |  |  |  |
|  |  |  |  |  |  |  |  |  |  |
| mean | 0.987345 | 0.126717 | 0.422954 | 0.814265 | 0.856381 | 0.906622 | 0.960938 | 0.944989 | 0.944085 |
| SEM | 0.004657 | 0.034566 | 0.086744 | 0.101163 | 0.047924 | 0.043171 | 0.039063 | 0.032392 | 0.037642 |

| **r Sol-r Tri** | Intact | 5dpo | 7dpo | 11dpo | 14dpo | 18dpo | 21dpo | 28dpo | 32dpo |
| --- | --- | --- | --- | --- | --- | --- | --- | --- | --- |
| 1 | 0.982759 | 0.384615 | 0.5625 | 0.654545 | 0.68 | 0.84 | 0.78125 | 0.693878 | 0.978261 |
| 2 | 1 | 0.465116 | 0.423077 | 0.6 | 0.95122 | 0.909091 | 1 | 0.833333 | 0.795918 |
| 3 | 1 | 0.544304 | 0.746479 | 0.787234 | 0.803922 | 0.774648 | 0.981982 | 0.977273 | 0.977273 |
| 4 | 0.946237 | 0.652174 | 0.710145 | 0.689655 | 0.977273 | 0.918367 | 0.983871 | 1 | 0.931507 |
| 5 | 0.969231 | 0.588235 | 0.583333 | 0.836735 | 0.75 | 1 |  | 1 | 1 |
| 6 | 1 | 0.543478 | 0.237288 | 0.97561 | 0.982456 | 0.942308 |  | 0.947368 |  |
| 7 | 1 |  | 0.838235 |  | 1 |  |  | 0.980198 |  |
| 8 | 0.975 |  | 0.82 |  | 0.966102 |  |  | 1 |  |
| 9 | 0.946429 |  | 0.965517 |  |  |  |  |  |  |
| 10 | 0.935897 |  | 0.881356 |  |  |  |  |  |  |
| 11 | 0.985714 |  | 0.672727 |  |  |  |  |  |  |
| 12 |  |  | 0.5 |  |  |  |  |  |  |
|  |  |  |  |  |  |  |  |  |  |
| mean | 0.976479 | 0.529654 | 0.661721 | 0.757297 | 0.888872 | 0.897402 | 0.936776 | 0.929006 | 0.936592 |
| SEM | 0.007297 | 0.038319 | 0.061718 | 0.056197 | 0.044099 | 0.032395 | 0.051999 | 0.038925 | 0.036896 |

The tables contain means of the fore-hindlimb coupling ratio (CR) defined as: the ratio of the number of EMG bursts of a forelimb muscle to the number of EMG bursts of a selected hindlimb muscle within a run, containing 10-15 forelimb cycles in individual rats, and the means±SEM calculated in the various groups of animals for particular time points. Abbreviations: dpo- days post spinal cord hemisection; **l Tri**, **r Tri** – left, right *Triceps Brachii* (forelimb extensor); **l Bic**, **r Bic** – left, right *Biceps* (forelimb flexor); **l Sol**, **r Sol** – left, right *Soleus* (hindlimb extensor); **l TA**, **r TA** – left, right *Tibialis Anterior* (hindlimb flexor).
