# Supplementary material for: Thoracic Hemisection in Rats Results in Initial Recovery Followed by a Late Decrement in Locomotor Movements, with Changes in Coordination Correlated with Serotonergic Innervation of the Ventral Horn
Source: PLoS One. 2015 Nov 25;10(11):e0143602. doi: 10.1371/journal.pone.0143602 (PMC4659566; doi:10.1371/journal.pone.0143602)
Supplement: S2 Table — The table contains means of r-value of intra- and interlimb established in Polar Plot analysis for individual rats and the means±SEM calculated in the various groups of animals for particular time points up to 4 weeks. Abbreviations: l-r TA—interlimb coordination established based on left—right TA EMG burst activity; l-r Sol—interlimb coordination established based on left—right Sol EMG burst activity; l Sol-l TA; r Sol-r TA—intralimb coordination established based on Sol versus TA in both hindlimbs separately. (DOCX) [file pone.0143602.s002.docx]

**S2 Table. The strength of inter- and intralimb coordination (EMG analysis).**

Inter-limb coordination

| **l-r TA** | Intact | 1wpo | 2wpo | 3wpo | 4wpo |
| --- | --- | --- | --- | --- | --- |
| 1 | 0.88901 | 0.36218 | 0.61258 | 0.71809 | 0.88682 |
| 2 | 0.90831 | 0.85415 | 0.97594 | 0.77125 | 0.5945 |
| 3 | 0.88349 | 0.78877 | 0.28398 | 0.9372 | 0.85393 |
| 4 | 0.85659 | 0.31714 | 0.38005 | 0.89171 | 0.80497 |
| 5 | 0.92698 | 0.99923 | 0.91944 | 0.94831 | 0.92385 |
| 6 | 0.82329 | 0.45767 | 0.8854 | 0.96441 | 0.95309 |
| 7 | 0.92937 | 0.09126 | 0.84854 | 0.9522 | 0.84922 |
| 8 |  |  |  |  | 0.88915 |
| 9 |  |  |  |  | 0.89602 |
|  |  |  |  |  |  |
| mean | 0.888149 | 0.552914 | 0.700847 | 0.88331 | 0.850172 |
| SEM | 0.014507 | 0.125331 | 0.105086 | 0.037282 | 0.035042 |

| **l-r Sol** | Intact | 1wpo | 2wpo | 3wpo | 4wpo |
| --- | --- | --- | --- | --- | --- |
| 1 | 0.92919 | 0.54343 | 0.58254 | 0.69179 | 0.94649 |
| 2 | 0.92513 | 0.78203 | 0.95832 | 0.78553 | 0.73081 |
| 3 | 0.89112 | 0.69605 | 0.39966 | 0.93477 | 0.92415 |
| 4 | 0.92302 | 0.76553 | 0.75244 | 0.95368 | 0.96566 |
| 5 | 0.88464 | 0.42989 | 0.91629 | 0.90096 | 0.92219 |
| 6 | 0.98 | 0.13388 | 0.83838 | 0.8588 | 0.94772 |
| 7 | 0.88621 | 0.21851 | 0.82045 | 0.92501 | 0.89533 |
| 8 |  |  |  |  | 0.89572 |
| 9 |  |  |  |  | 0.94541 |
|  |  |  |  |  |  |
| mean | 0.917044 | 0.509903 | 0.752583 | 0.864363 | 0.908164 |
| SEM | 0.012803 | 0.098546 | 0.074732 | 0.03585 | 0.023556 |

Intra-limb coordination

| **l Sol-l TA** | Intact | 1wpo | 2wpo | 3wpo | 4wpo |
| --- | --- | --- | --- | --- | --- |
| 1 | 0.98688 | 0.97103 | 0.84321 | 0.94237 | 0.95431 |
| 2 | 0.91366 | 0.67758 | 0.97804 | 0.8498 | 0.90419 |
| 3 | 0.96124 | 0.57038 | 0.93401 | 0.95567 | 0.90349 |
| 4 | 0.97091 | 0.33421 | 0.62332 | 0.98451 | 0.92206 |
| 5 | 0.96195 | 0.32005 | 0.89814 | 0.97828 | 0.96498 |
| 6 | 0.89367 | 0.03905 | 0.8868 | 0.83514 | 0.93843 |
| 7 | 0.93188 | 0.16719 | 0.87833 | 0.94961 | 0.92126 |
| 8 |  |  |  |  | 0.88154 |
| 9 |  |  |  |  | 0.96386 |
|  |  |  |  |  |  |
| Mean | 0.945741 | 0.439927 | 0.863121 | 0.927911 | 0.928236 |
| SEM | 0.012688 | 0.121126 | 0.043136 | 0.022834 | 0.009762 |

| **r Sol-r TA** | Intact | 1wpo | 2wpo | 3wpo | 4wpo |
| --- | --- | --- | --- | --- | --- |
| 1 | 0.90834 | 0.9357 | 0.91529 | 0.94265 | 0.9088 |
| 2 | 0.9183 | 0.92427 | 0.96353 | 0.91508 | 0.89217 |
| 3 | 0.90821 | 0.86126 | 0.86362 | 0.96349 | 0.95784 |
| 4 | 0.88528 | 0.95291 | 0.83602 | 0.96753 | 0.87708 |
| 5 | 0.92902 | 0.84924 | 0.95968 | 0.87454 | 0.92497 |
| 6 | 0.92259 | 0.92228 | 0.91208 | 0.92405 | 0.95329 |
| 7 | 0.93397 | 0.90218 | 0.88618 | 0.92869 | 0.93749 |
| 8 |  |  |  |  | 0.91314 |
| 9 |  |  |  |  | 0.90343 |
|  |  |  |  |  |  |
| Mean | 0.915101 | 0.906834 | 0.9052 | 0.930861 | 0.91869 |
| SEM | 0.006169 | 0.014569 | 0.017859 | 0.011973 | 0.009067 |

The table contains means of ***r***-value of intra- and interlimb established in Polar Plot analysis for individual rats and the means±SEM calculated in the various groups of animals for particular time points up to 4 weeks. Abbreviations: **l-r TA** - interlimb coordination established based on left – right TA EMG burst activity**; l-r Sol**  - interlimb coordination established based on left – right Sol EMG burst activity; **l Sol-l TA; r Sol-r TA -** intralimb coordination established based on Sol versus TA in both hindlimbs separately; wpo- weeks post spinal cord hemisection.
