# Supplementary material for: Thoracic Hemisection in Rats Results in Initial Recovery Followed by a Late Decrement in Locomotor Movements, with Changes in Coordination Correlated with Serotonergic Innervation of the Ventral Horn
Source: PLoS One. 2015 Nov 25;10(11):e0143602. doi: 10.1371/journal.pone.0143602 (PMC4659566; doi:10.1371/journal.pone.0143602)
Supplement: S3 Table — The table contains means of phase shifts between onsets of EMG bursts in specific muscles for intra- and interlimb coordination in individual rats and the means±SEM calculated in the various groups of animals for particular time points. Abbreviations: l-r TA—interlimb coordination established based on left—right TA EMG burst activity; l-r Sol—interlimb coordination established based on left—right Sol EMG burst activity; l Sol-l TA; r Sol-r TA—intralimb coordination established based on Sol versus TA in both hindlimbs separately. (DOCX) [file pone.0143602.s003.docx]

**S3 Table. The phase shift of inter- and intralimb coordination (EMG analysis).**

Inter-limb coordination

| **l-r TA** | Intact | 2 wpo | 3 wpo | 1mpo | 2mpo | 3mpo | 5mpo |
| --- | --- | --- | --- | --- | --- | --- | --- |
| 1 | 166.84 | 109.364 | 118.406 | 140.986 | 176.397 | 189.58 | 150.928 |
| 2 | 192.978 | 150.224 | 150.144 | 111.604 | 129.559 | 145.489 | 143.485 |
| 3 | 192.257 | 129.806 | 130.761 | 134.89 | 139.158 | 159.79 | 172 |
| 4 | 171.394 | 135.51 | 120.956 | 175.659 | 163.23 | 153.018 | 140.15 |
| 5 | 202.284 | 130.563 | 120.395 | 160.078 | 159.485 | 166.927 |  |
| 6 | 174.776 |  | 159.576 | 119.839 |  | 159.549 |  |
| 7 | 187.669 |  |  | 143.769 |  |  |  |
|  |  |  |  |  |  |  |  |
| mean | 184.0283 | 131.0934 | 133.373 | 140.975 | 153.5658 | 162.3922 | 151.6408 |
| SEM | 4.964162 | 6.555882 | 7.121113 | 8.341126 | 8.466429 | 6.186517 | 7.150528 |

| **l-r Sol** | Intact | 2 wpo | 3 wpo | 1mpo | 2mpo | 3mpo | 5mpo |
| --- | --- | --- | --- | --- | --- | --- | --- |
| 1 | 193.808 | 113.38 | 105.277 | 144.575 | 101.908 | 176.086 | 142.678 |
| 2 | 171.351 | 110.314 | 152.381 | 138.713 | 157.894 | 168.433 | 141.477 |
| 3 | 190.207 | 164.274 | 133.964 | 149.284 | 151.841 | 145.134 | 137.328 |
| 4 | 167.089 | 161.065 | 140.556 | 161.761 | 157.664 | 157.785 | 155.38 |
| 5 | 192.269 | 135.788 | 170.147 | 152.464 | 125.545 | 130.402 |  |
| 6 | 179.26 |  | 155.867 | 140.465 |  | 152.443 |  |
| 7 | 183.309 |  |  | 139.087 |  |  |  |
|  |  |  |  |  |  |  |  |
| mean | 182.4704 | 136.9642 | 143.032 | 146.6213 | 138.9704 | 155.0472 | 144.2158 |
| SEM | 3.951031 | 11.3897 | 9.130833 | 3.206511 | 11.01605 | 6.687664 | 3.893873 |

Intra-limb coordination

| **l Sol-l TA** | Intact | 2 wpo | 3 wpo | 1mpo | 2mpo | 3mpo | 5mpo |
| --- | --- | --- | --- | --- | --- | --- | --- |
| 1 | 118.967 | 120.017 | 111.268 | 88 | 153.794 | 110.364 | 89.9937 |
| 2 | 118.532 | 96.9836 | 104.774 | 127.3 | 102.33 | 100.201 | 102.61 |
| 3 | 110.843 | 82.1553 | 90.5682 | 98.0999 | 88.3081 | 97.52 | 116.112 |
| 4 | 109.069 | 71.8558 | 115.812 | 117.735 | 111.263 | 113.588 | 75.97 |
| 5 | 163.071 | 117.599 | 89.8906 | 106.734 | 132.497 | 131.506 |  |
| 6 | 149.168 |  | 126.939 | 90.6408 |  | 96.632 |  |
| 7 | 148.396 |  |  | 132.759 |  |  |  |
|  |  |  |  |  |  |  |  |
| mean | 131.1494 | 97.72214 | 106.542 | 108.7527 | 117.6384 | 108.3018 | 96.17143 |
| SEM | 8.235532 | 9.497605 | 5.9438 | 6.685555 | 11.53964 | 5.442689 | 8.589449 |

| **r Sol-r TA** | Intact | 2 wpo | 3 wpo | 1mpo | 2mpo | 3mpo | 5mpo |
| --- | --- | --- | --- | --- | --- | --- | --- |
| 1 | 140.838 | 102.633 | 92.372 | 80.7059 | 88.1923 | 97.4692 | 77.9512 |
| 2 | 98.0634 | 54.8346 | 105.29 | 153.235 | 128.432 | 123.293 | 101.281 |
| 3 | 107.345 | 114.139 | 95.0413 | 113.321 | 100.497 | 80.9975 | 77.11 |
| 4 | 104.716 | 91.2691 | 135.686 | 102.205 | 105.884 | 117.868 | 90.75 |
| 5 | 150.541 | 118.138 | 136.575 | 96.234 | 97.7599 | 94.59 |  |
| 6 | 152.343 |  | 122.794 | 109.808 |  | 88.34 |  |
| 7 | 142.552 |  |  | 126.831 |  |  |  |
|  |  |  |  |  |  |  |  |
| mean | 128.0569 | 96.20274 | 114.6264 | 111.7628 | 104.153 | 100.4263 | 86.77305 |
| SEM | 8.920555 | 11.35755 | 8.078697 | 8.797009 | 6.713599 | 6.815332 | 5.755406 |

The table contains means of phase shifts between onsets of EMG bursts in specific muscles for intra- and interlimb coordination in individual rats and the means±SEM calculated in the various groups of animals for particular time points. Abbreviations: **l-r TA** - interlimb coordination established based on left – right TA EMG burst activity**; l-r Sol**  - interlimb coordination established based on left – right Sol EMG burst activity; **l Sol-l TA; r Sol-r TA -** intralimb coordination established based on Sol versus TA in both hindlimbs separately; wpo- weeks; mpo- months post spinal cord hemisection.
