# Supplementary material for: Thoracic Hemisection in Rats Results in Initial Recovery Followed by a Late Decrement in Locomotor Movements, with Changes in Coordination Correlated with Serotonergic Innervation of the Ventral Horn
Source: PLoS One. 2015 Nov 25;10(11):e0143602. doi: 10.1371/journal.pone.0143602 (PMC4659566; doi:10.1371/journal.pone.0143602)
Supplement: S4 Table — The table contains means of hindlimb BOS in individual rats and the means±SEM calculated in the various groups of animals for particular time points. (DOCX) [file pone.0143602.s004.docx]

**S4 Table. Results of CatWalk analysis showing the Base of Support (BOS).**

|  | Intact | 2 wpo | 1mpo | 2mpo | 3mpo | 6mpo |
| --- | --- | --- | --- | --- | --- | --- |
| 1 | 32.12667 | 47.628 | 53.00167 | 53.14625 | 59.28625 | 67.92714 |
| 2 | 32.21 | 40.904 | 34.94857 | 39.75571 | 46.86444 | 46.20333 |
| 3 | 35.56625 | 57.144 | 43.03 | 36.4325 | 41.81143 | 48.325 |
| 4 | 37.7175 | 59.6375 | 43.92667 | 45.80857 | 46.22333 | 47.895 |
| 5 | 42.12 | 49.206 | 34.555 | 48.58 | 47.945 |  |
| 6 | 32.71 | 47.215 | 40.26 | 38.116 | 61.854 |  |
| 7 | 30.47231 | 55.05333 | 60.598 | 65.082 |  |  |
| 8 | 35.344 |  |  |  |  |  |
| 9 | 34.497 |  |  |  |  |  |
|  |  |  |  |  |  |  |
| mean | 34.75153 | 50.96969 | 44.33142 | 46.703 | 50.66408 | 52.58762 |
| SEM | 1.17851 | 2.48626 | 3.59174 | 3.81448 | 3.263313 | 5.133636 |

The table contains means of hindlimb BOS in individual rats and the means±SEM calculated in the various groups of animals for particular time points. Abbreviations: wpo- weeks; mpo- months post spinal cord hemisection.
