# Supplementary material for: Thoracic Hemisection in Rats Results in Initial Recovery Followed by a Late Decrement in Locomotor Movements, with Changes in Coordination Correlated with Serotonergic Innervation of the Ventral Horn
Source: PLoS One. 2015 Nov 25;10(11):e0143602. doi: 10.1371/journal.pone.0143602 (PMC4659566; doi:10.1371/journal.pone.0143602)
Supplement: S5 Table — The table contains means of HA for left and right hindlimbs in individual rats and the means±SEM calculated in the various groups of animals for particular time points. Abbreviations: RF- right forelimb; RH–right hindlimb; LF- left forelimb; LH–left hindlimb. (DOCX) [file pone.0143602.s005.docx]

**S5 Table. Results of CatWalk analysis showing the abduction of hindlimbs (HA)**.

| **RH** | Intact | 2 wpo | 1mpo | 2mpo | 3mpo | 5mpo |
| --- | --- | --- | --- | --- | --- | --- |
| 1 | 7.172131 | 12.61375 | 12.2799 | 13.44002 | 8.196721 | 12.85268 |
| 2 | 8.490437 | 12.33607 | 11.85012 | 12.85974 | 8.460838 | 9.818745 |
| 3 | 9.335155 | 11.63934 | 10.40036 | 9.972678 | 10.74031 | 16.97325 |
| 4 | 4.371585 | 8.540984 | 11.834 | 13.6612 | 12.73484 | 17.6929 |
| 5 | 5.614754 | 12.48634 |  | 10.11787 | 18.36778 |  |
| 6 | 8.025956 |  |  |  | 18.2024 |  |
| 7 | 4.59114 |  |  |  |  |  |
| 8 | 6.002879 |  |  |  |  |  |
| 9 | 6.451991 |  |  |  |  |  |
| 10 | 10.53673 |  |  |  |  |  |
| 11 | 9.861667 |  |  |  |  |  |
|  |  |  |  |  |  |  |
| mean | 7.314039 | 11.5233 | 11.59109 | 12.0103 | 12.78381 | 14.3344 |
| SEM | 0.634565 | 0.764356 | 0.410123 | 0.813153 | 1.865919 | 1.84458 |

| **LH** | Intact | 2 wpo | 1mpo | 2mpo | 3mpo | 5mpo |
| --- | --- | --- | --- | --- | --- | --- |
| 1 | 5.344945 | 15.38251 | 11.09897 | 8.688525 | 15.38251 | 16.51054 |
| 2 | 6.803279 | 12.84153 | 10.48087 | 12.54098 | 14.01639 | 10.40301 |
| 3 | 5.464481 | 9.836066 | 7.470726 | 13.74317 | 9.049787 | 13.63858 |
| 4 | 5.464481 | 10.81967 | 11.79333 | 5.737705 | 14.59933 | 9.93719 |
| 5 | 8.469945 | 13.0929 |  | 13.4688 | 8.4668 |  |
| 6 | 4.571949 |  |  |  |  |  |
| 7 | 6.970628 |  |  |  |  |  |
| 8 | 9.62139 |  |  |  |  |  |
| 9 | 6.713505 |  |  |  |  |  |
| 10 | 6.913333 |  |  |  |  |  |
| 11 | 8.446467 |  |  |  |  |  |
|  |  |  |  |  |  |  |
| mean | 6.798582 | 12.39454 | 10.21098 | 10.83584 | 12.30297 | 12.62233 |
| SEM | 0.468633 | 0.96544 | 0.951937 | 1.563562 | 1.466153 | 1.535315 |

The table contains means of HA for left and right hindlimbs in individual rats and the means±SEM calculated in the various groups of animals for particular time points. Abbreviations: **RF**- right forelimb**; RH –** right hindlimb**; LF-** left forelimb**; LH –** left hindlimb; wpo- weeks; mpo- months post spinal cord hemisection.
