# Supplementary material for: Thoracic Hemisection in Rats Results in Initial Recovery Followed by a Late Decrement in Locomotor Movements, with Changes in Coordination Correlated with Serotonergic Innervation of the Ventral Horn
Source: PLoS One. 2015 Nov 25;10(11):e0143602. doi: 10.1371/journal.pone.0143602 (PMC4659566; doi:10.1371/journal.pone.0143602)
Supplement: S6 Table — The table contains means of RPD between left and right fore—hindlimb pairs in individual rats and the means±SEM calculated in the various groups of animals for particular time points. Abbreviations: RH–right hindlimb; LH–left hindlimb; wpo- weeks; mpo- months post spinal cord hemisection. (DOCX) [file pone.0143602.s006.docx]

**S6 Table. Results of CatWalk analysis showing the Relative Print Distance (RPD)**.

| **RF-RH** | Intact | 2 wpo | 1mpo | 2mpo | 3mpo | 5mpo |
| --- | --- | --- | --- | --- | --- | --- |
| 1 | -6.27049 | 9.794269 | 2.978142 | 12.42096 | 19.71442 | 24.54277 |
| 2 | 1.714481 | 1.759953 | 18.45043 | 3.4 | 3.966302 | 14.3184 |
| 3 | -2.56148 | 18.27869 | 7.750084 | 12.0739 | 21.9555 | 2.764 |
| 4 | -2.06056 | -0.03279 | 6.74 | 1.571038 | 12.08561 | 39.797 |
| 5 | -8.07377 | 2.991803 | 27.366 | -0.614 | -3.885 |  |
| 6 | 9.157884 |  |  | 33.692 | 23.68833 |  |
| 7 | 8.786349 |  |  |  |  |  |
| 8 | 0.709285 |  |  |  |  |  |
| 9 | -3.28845 |  |  |  |  |  |
| 10 | -5.039 |  |  |  |  |  |
| 11 | -11.797 |  |  |  |  |  |
|  |  |  |  |  |  |  |
| mean | -1.70207 | 6.558385 | 12.65693 | 10.42398 | 12.92086 | 20.35554 |
| SEM | 1.961744 | 3.369165 | 4.487134 | 5.156416 | 4.498275 | 7.860304 |

| **LF-LH** | Intact | 2 wpo | 1mpo | 2mpo | 3mpo | 5mpo |
| --- | --- | --- | --- | --- | --- | --- |
| 1 | -6.07923 | 5.135474 | 7.789921 | 12.24727 | 17.53643 | 25.121 |
| 2 | 1.895492 | -3.37432 | 14.65574 | 8.5 | 2.472678 | 16.94526 |
| 3 | -4.43989 | 15.47131 | 6.533958 | 4.790528 | 20.37341 | 13.372 |
| 4 | -3.9071 | -9.65847 | 18.916 | 1.967213 | 13.90559 | 33.318 |
| 5 | -8.16257 | -3.12568 | 24.766 | 1.644 | 2.508333 |  |
| 6 | 10.68473 |  |  | 27.49 | 23.92667 |  |
| 7 | 5.28347 |  |  |  |  |  |
| 8 | -2.58343 |  |  |  |  |  |
| 9 | -1.42467 |  |  |  |  |  |
| 10 | -0.45 |  |  |  |  |  |
| 11 | -3.549 |  |  |  |  |  |
|  |  |  |  |  |  |  |
| mean | -1.15747 | 0.889663 | 14.53232 | 9.439835 | 13.45385 | 22.18906 |
| SEM | 1.626482 | 4.337159 | 3.416093 | 3.969906 | 3.718522 | 4.450539 |

The table contains means of RPD between left and right fore – hindlimb pairs in individual rats and the means±SEM calculated in the various groups of animals for particular time points.

Abbreviations: **RH –** right hindlimb**; LH –** left hindlimb; wpo- weeks; mpo- months post spinal cord hemisection.
