# Supplementary material for: Thoracic Hemisection in Rats Results in Initial Recovery Followed by a Late Decrement in Locomotor Movements, with Changes in Coordination Correlated with Serotonergic Innervation of the Ventral Horn
Source: PLoS One. 2015 Nov 25;10(11):e0143602. doi: 10.1371/journal.pone.0143602 (PMC4659566; doi:10.1371/journal.pone.0143602)
Supplement: S7 Table — The table contains means of V in individual rats and the means±SEM calculated in the various groups of animals for particular time points. Abbreviations: wpo- weeks; mpo- months post spinal cord hemisection. (DOCX) [file pone.0143602.s007.docx]

**S7 Table.** **Results of CatWalk analysis showing the speed of locomotion (V)**.

|  | Intact | 2 wpo | 1mpo | 2mpo | 3mpo | 6mpo |
| --- | --- | --- | --- | --- | --- | --- |
| 1 | 64.12049 | 51.39167 | 38.1415 | 49.60113 | 37.18048 | 31.65164 |
| 2 | 70.17019 | 51.52034 | 38.06782 | 39.24363 | 43.05 | 24.20466 |
| 3 | 61.30448 | 37.61212 | 26.07765 | 31.82991 | 42.32041 | 25.86512 |
| 4 | 51.50746 | 36.47143 | 36.99966 | 33.19856 | 33.62664 | 28.5675 |
| 5 | 60.3125 | 42.40078 | 29.22822 | 31.13816 | 48.85227 |  |
| 6 | 47.14247 | 26.72881 | 36.34076 | 31.05787 | 36.80478 |  |
| 7 | 50.92419 | 21.77291 | 37.27566 | 32.86131 |  |  |
| 8 | 60.76458 |  |  |  |  |  |
| 9 | 54.34698 |  |  |  |  |  |
|  |  |  |  |  |  |  |
|  |  |  |  |  |  |  |
|  |  |  |  |  |  |  |
| mean | 57.84371 | 38.27115 | 34.59018 | 35.56151 | 40.30576 | 27.57223 |
| SEM | 2.451516 | 4.29239 | 1.838747 | 2.56959 | 2.245495 | 1.630105 |

The table contains means of V in individual rats and the means±SEM calculated in the various groups of animals for particular time points. Abbreviations: wpo- weeks; mpo- months post spinal cord hemisection.
