# Supplementary material for: Thoracic Hemisection in Rats Results in Initial Recovery Followed by a Late Decrement in Locomotor Movements, with Changes in Coordination Correlated with Serotonergic Innervation of the Ventral Horn
Source: PLoS One. 2015 Nov 25;10(11):e0143602. doi: 10.1371/journal.pone.0143602 (PMC4659566; doi:10.1371/journal.pone.0143602)
Supplement: S8 Table — The table contains means of pixels established for individual rats in the left and right ventral horns of their spinal cords and the means±SEM in the various groups of animals for particular time points. Abbreviations: wpo- weeks; mpo- months post spinal cord hemisection. (DOCX) [file pone.0143602.s008.docx]

**S8 Table. The length of serotonergic fibers on ipsilateral and contralateral sides of the spinal cord.**

| **Contralateral** | intact | 1wpo | 2wpo | 3wpo | 4wpo | 3mpo | 6mpo |
| --- | --- | --- | --- | --- | --- | --- | --- |
| 1 | 21589.92 | 29504.14 | 13609.07 | 15949.45 | 9049.213 | 28734.49 | 33792.06 |
| 2 | 28989.61 | 5994.174 | 9732.2 | 10078.68 | 28514.11 | 37623.4 | 11567.79 |
| 3 | 29033.82 | 432.32 | 19308.93 | 5611.625 | 37883.89 | 21572.52 | 23794.23 |
| 4 | 21607.17 | 4479.163 | 2969.098 | 16979.14 | 27866.75 | 36099.82 | 22933.76 |
| 5 | 26285.07 | 8759.994 | 10144.97 | 20435.21 | 27546.82 | 36738.46 | 15356.45 |
| 6 | 27223.91 | 29056.87 | 16946.75 | 9493.81 | 32799.01 | 34887.72 | 22790.61 |
| 7 | 34711.16 | 8449.713 | 13641.76 | 19812.49 | 24600.32 | 25822.75 | 28025.21 |
| 8 | 50779.95 | 6926.684 | 12943.35 | 27116.82 | 24600.32 | 25822.75 | 23896.5 |
|  |  |  |  |  |  |  |  |
| mean | 30027.58 | 11700.38 | 12412.02 | 15684.65 | 26607.55 | 30912.74 | 22769.58 |
| SEM | 3323.687 | 3945.835 | 1756.866 | 2475.835 | 2951.833 | 2177.313 | 2434.493 |

| **Ipsilateral** | intact | 1wpo | 2wpo | 3wpo | 4wpo | 3mpo | 6mpo |
| --- | --- | --- | --- | --- | --- | --- | --- |
| 1 | 21589.92 | 225.603 | 819.698 | 1721.967 | 4681.735 | 902.835 | 1714.129 |
| 2 | 28989.61 | 766.978 | 672.936 | 312.158 | 933.429 | 1298.811 | 2326.986 |
| 3 | 29033.82 | 379.192 | 1241.634 | 377.535 | 1364.501 | 789.105 | 2015.469 |
| 4 | 21607.17 | 61.507 | 1273.894 | 1232.221 | 903.606 | 2771.337 | 2041.683 |
| 5 | 26285.07 | 225.189 | 942.252 | 2444.64 | 1190.067 | 1637.214 | 586.332 |
| 6 | 27223.91 | 88.788 | 729.179 | 808.122 | 1277.935 | 1342.173 | 450.436 |
| 7 | 34711.16 | 555.155 | 2039.623 | 694.454 | 1190.067 | 1465.775 | 1850.338 |
| 8 | 50779.95 | 147.779 | 550.352 | 694.454 | 1277.935 | 1962.739 | 3062.169 |
|  |  |  |  |  |  |  |  |
| mean | 30027.58 | 306.274 | 1033.696 | 1035.694 | 1602.409 | 1521.249 | 1755.943 |
| SEM | 3323.687 | 87.108 | 170.267 | 258.294 | 443.713 | 222.573 | 306.603 |

The table contains means of pixels established for individual rats in the left and right ventral horns of their spinal cords and the means±SEM in the various groups of animals for particular time points. Abbreviations: wpo- weeks; mpo- months post spinal cord hemisection.
