# Supplementary material for: Thoracic Hemisection in Rats Results in Initial Recovery Followed by a Late Decrement in Locomotor Movements, with Changes in Coordination Correlated with Serotonergic Innervation of the Ventral Horn
Source: PLoS One. 2015 Nov 25;10(11):e0143602. doi: 10.1371/journal.pone.0143602 (PMC4659566; doi:10.1371/journal.pone.0143602)
Supplement: S9 Table — The table contains coefficient of correlation and its significance for the relationships between various locomotor parameters and the serotonergic fiber lengths in the left and right ventral horns of their spinal cords. Abbreviations: r-value—the strength of intra- or interlimb coordination, CC—correlation coefficient, p–significance of the CC (DOCX) [file pone.0143602.s009.docx]

**S9 Table. Correlation between ipsilateral or contralateral serotonergic fiber lengths and locomotor parameters.**

| Parameter | | Ipsilateral 5HT length | | Contralatera 5HT length | |
| --- | --- | --- | --- | --- | --- |
|  |  | CC | *p* | CC | *p* |
| ***r***-value | r TA-r Sol | 0.588 | 0.219 | 0.591 | 0.217 |
|  | l TA-l Sol | 0.976 | 0.001** | 0.767 | 0.075 |
|  | l-r TA | 0.782 | 0.066 | 0.802 | 0.055 |
|  | l-r Sol | 0.948 | 0.004** | 0.866 | 0.026* |
| Phase shift | r TA-r Sol | 0.684 | 0.134 | 0.460 | 0.369 |
|  | l TA-l Sol | -0.644 | 0.168 | -0,451 | 0.370 |
|  | l-r TA | 0.051 | 0.102 | 0.868 | 0.056 |
|  | l-r Sol | 0.045 | 0.093 | 0.397 | 0.508 |
| CatWalk | BOS | -0.186 | 0.814 | -0.298 | 0.702 |
|  | rHA | 0.605 | 0.395 | 0.294 | 0.706 |
|  | lHA | -0.260 | 0.740 | -0.312 | 0.688 |
|  | rRPD | 0.852 | 0.148 | 0.496 | 0.504 |
|  | lRPD | 0.901 | 0.495 | 0.660 | 0.340 |
|  | V | -0.498 | 0.502 | 0.038 | 0.962 |

The table contains coefficient of correlation and its significance for the relationships between various locomotor parameters and the serotonergic fiber lengths in the left and right ventral horns of their spinal cords. Abbreviations: ***r***-value – the strength of intra- or interlimb coordination, CC – correlation coefficient, *p* – significance of the CC.
